# Structural and biophysical characterization of *Staphylococcus aureus Sa*MazF shows conservation of functional dynamics

**DOI:** 10.1093/nar/gku266

**Published:** 2014-04-19

**Authors:** Valentina Zorzini, Lieven Buts, Mike Sleutel, Abel Garcia-Pino, Ariel Talavera, Sarah Haesaerts, Henri De Greve, Ambrose Cheung, Nico A. J. van Nuland, Remy Loris

**Affiliations:** 1 Structural Biology Brussels, Vrije Universiteit Brussel, Pleinlaan 2, 1050 Brussels, Belgium; 2 Molecular Recognition Unit, Department of Structural Biology, VIB, Pleinlaan 2, 1050 Brussels, Belgium; 3 Department of Microbiology and Immunology, Dartmouth Medical School, Hanover, NH 03755, USA

## Abstract

The *Staphylococcus aureus* genome contains three toxin–antitoxin modules, including one *mazEF* module, Sa*mazEF*. Using an on-column separation protocol we are able to obtain large amounts of wild-type *Sa*MazF toxin. The protein is well-folded and highly resistant against thermal unfolding but aggregates at elevated temperatures. Crystallographic and nuclear magnetic resonance (NMR) solution studies show a well-defined dimer. Differences in structure and dynamics between the X-ray and NMR structural ensembles are found in three loop regions, two of which undergo motions that are of functional relevance. The same segments also show functionally relevant dynamics in the distantly related CcdB family despite divergence of function. NMR chemical shift mapping and analysis of residue conservation in the MazF family suggests a conserved mode for the inhibition of MazF by MazE.

## INTRODUCTION

Pathogenic bacteria are adept at responding to environmental changes. Chromosomal toxin–antitoxin (TA) modules are thought to facilitate these responses by altering gene transcription and translation. TA modules are small operons encoding two proteins: a ‘toxin’ that interferes with basic cellular metabolism, usually translation or transcription, and an ‘antitoxin’ that neutralizes the toxin and protects the cell from its potentially destructive activity (for reviews see 1–4).

TA modules are activated upon environmental stress (e.g. antibiotics or nutritional stress) through proteolytic degradation of the antitoxin (5–10). Under normal growth conditions, the antitoxin and toxin genes are transcribed and translated together, thus leading to the formation of an inert TA complex. This complex also acts as an auto-repressor, limiting the number of TA proteins present in the cytoplasm via a mechanism termed ‘conditional cooperativity’ (11–14). Several unrelated families of TA modules exist that differ in terms of amino acid sequence and biochemical activities of the toxin. The latter include ribosome-dependent and ribosome-independent degradation of mRNA (15–19), phosphorylation of elongation factor Tu and glutamyl-tRNA synthetase (19,20), or poisoning of gyrase (21–26).

The *mazEF* module was initially discovered on plasmids R1 and R100 where it was termed *kis/kid* and *pemIK*, respectively, and contributes to plasmid stability (27,28). It was the first so-called plasmid addiction system for which homologues were discovered in bacterial chromosomes (29,30). Subsequent bioinformatics analyses have shown that the *mazEF* family is widely distributed in the genomes of both Gram-negative and Gram-positive bacteria, but seems to be absent in Archeae (31–34). The toxin MazF is activated under a number of stressful conditions via proteolytic degradation of its neutralizing antitoxin MazE by the ClpPA or Lon proteases, (18,30,35) and was proposed to be under control of quorum sensing (36). Prolonged over-expression of MazF leads to cell death (37).

*Escherichia coli* MazF (*Ec*MazF) was shown to degrade mRNA in a sequence-specific manner without the requirement of the mRNA being bound to the ribosome or actively being translated (17,35,38). This activity was later confirmed for a number of family members from different organisms or plasmids and it was shown that the exact RNA cleavage specificity may vary, although most (but not all) identified cutting sequences contain an ACA motif (39–44). The RNase activity of MazF proteins was proposed to result in selective degradation of the cellular pool of mRNAs, leading to a shift in the expression profile toward a subset of proteins (45–47). Later on, it was demonstrated that *Ec*MazF also cuts ribosomal RNA, and that the resulting modified ribosomes specifically translate leader-less mRNA that also results from MazF-specific mRNA cleavage (48,49). Recently, evidence was presented that a MazF homolog from *Mycobacterium* halts translation through cleavage of the 23S rRNA (50).

TA modules including *mazEF* modules have been well studied in Gram-negative bacteria, in particular *E. coli* and *Mycobacterium* *tuberculosis*. Next to ‘classic’ *mazEF* modules where both toxin and antitoxin can be clearly identified as MazF and MazE family members (e.g. *Bacillus subtilis*; (43)), Gram-positive bacteria also contain variants type of *mazEF* modules where the antitoxin is unusually short and possibly unrelated to the classic MazE proteins. This is the case of the sole *mazEF* module found in the chromosomes of several *Staphylococcus* species including MRSA strains (51). Transcription regulation and activation of *Staphylococcus aureus mazEF* (Sa*mazEF*) differs from what is observed in Gram-negative bacteria (52). Rather than being autoregulated as is usually observed in TA modules, Sa*mazEF* is linked to the *sigB* operon that is located immediately downstream and with which it is co-transcribed. In addition, the transcription regulator SarA binds and activates the Sa*mazEF* promoter.

In this paper, we present a method to obtain large quantities of active *Sa*MazF and provide the structure of this protein as determined by nuclear magnetic resonance (NMR) and X-ray crystallography. The structural and dynamic properties of *Sa*MazF are compared to its *E. coli* and *B. subtilis* counterparts as well as to CcdB family members, which adopt the same fold but function as gyrase poisons rather than ribonucleases.

## MATERIALS AND METHODS

### Cloning, expression and purification of *Sa*MazF

The cloning and expression of the *samazE* and *samazF* genes was described previously (53,54). Cells were grown in unlabeled LB medium or in ^13^C^15^N-enriched minimal medium (SPECTRA 9 from Cambridge Isotope Laboratories). The cells were harvested by centrifugation for 25 min at 5500 rpm with Beckman JLA 81000 rotor and the pellet was resuspended in 50 ml of lysis buffer (100 mM Tris-HCl pH 8.0, 1 M NaCl, 20 mM imidazole, 0.1 mg/ml AESBF and 1 μg/ml leupeptin, DNase I 50 μg/ml, MgCl_2_ 20 mM). The cell suspension was lysed by passing it twice through a cell cracker (10 000–15 000 psi) and subsequently centrifuged for 30 min at 18 000 rpm (Beckman JA-20 rotor).

The supernatant was filtered through a 45 μm filter and loaded on a pre-packed column of 1 ml Ni-NTA resin (Qiagen) pre-equilibrated with 10 column volumes of washing buffer (20 mM Tris-HCl pH 7.0, 300 mM NaCl, 20 mM imidazole). The column was further washed with the same buffer until the OD^280 nm^ stabilizes. Subsequently, a linear (0–3 M over 15 column volumes) guanidinium hydrochloride (GdHCl) gradient is applied in 50 mM Tris-HCl pH 7.0, 500 mM NaCl, which elutes *Sa*MazE. The column is further washed with 5 column volumes of the same GdHCl-containing buffer, after which the GdHCl concentration is linearly decreased while at the same time adding a 0–1 M imidazole gradient in the same buffer.

*Sa*MazF elutes in 2.75 M GdHCl. The *Sa*MazF-containing fractions were diluted using refolding buffer (50 mM Tris-HCl pH 7.0, 500 mM NaCl, 500 mM L-Arg) to obtain a final concentration of 0.2 M GdHCl. The protein solution was subsequently dialyzed against this refolding buffer for two times 4 h at 277 K. The protein solution was then dialyzed overnight in 20 mM Tris-HCl pH 7.0, 250 mM NaCl.

In a last polishing step, *Sa*MazF is loaded on a Superdex 75PG 16/90 column equilibrated with 20 mM Tris-HCl pH 7.0, 250 mM NaCl to remove any remaining contaminants. The purity of the sample was determined by SDS-PAGE analysis in presence of β-mercaptoethanol. *Sa*MazF concentrations were determined spectrophotometrically by measuring the absorbance at 280 nm using a theoretical extinction coefficient of 5960 M^−1^ cm^−1^ calculated from the amino acid sequence according to (55).

### *In vitro* ribonuclease assay

Bacteriophage MS2 genomic RNA (10 mM Tris-HCl pH 7.0, 1.0 mM EDTA) was obtained from Roche Applied Science. Mixtures of 0.25 μl of RNA (0.8 μg/μl), 2.5 μl or 5 μl of *Sa*MazF, 5 μl of *Sa*MazE or 2.5 μl *Sa*MazF + 5 μl of *Sa*MazE (final concentration of 1 μM or 2 μM *Sa*MazF, 1 μM *Sa*MazE or 1 μM *Sa*MazF + 5 μM *Sa*MazE) in a 10 μl final reaction volume (buffer: 20 mM Tris-HCl pH 7.0, 75 mM NaCl) were incubated at 37°C for 1 h. Samples were loaded on a 6% polyacrylamide gel containing 7 M urea. The gel was stained in water and ethidium bromide. The low range ssRNA ladder of 50, 80, 150, 300, 500, 1000 bases was bought from New England Biolabs Inc.

### *In vivo* activity assay

Non-tagged, N-terminal and C-terminal his-tagged *SamazF* sequences were cloned under control of the Plac promoter in a pTrc99a expression plasmid. These constructs were transformed in *E. coli* strain DH5α and plated on LB medium supplemented with 0.2% glucose. Transformants were tested for *in vivo* activity by streaking the same colonies on LB medium with glucose and LB medium with isopropyl β-D-thiogalactopyranoside (1 μM) to induce the Plac promoter. Non-growing colonies after IPTG induction were considered producing active *Sa*MazF.

### Mass spectrometry

Purified *Sa*MazF was extensively dialyzed against water and subsequently further desalted and concentrated using C18 spin columns (Thermo Scientific) according to the manufacturer's instructions except that proteins were eluted with 60 μl of 70% acetonitrile in water containing 0.1% formic acid (v/v). Hundred microliters of this *Sa*MazF sample was further diluted using a 50:50 acetonitrile/water mixture containing 0.1% (v/v) formic acid to an approximate final concentration of 5 μM.

The sample was introduced by off-line infusion using a capillary electrospray at 1.5 μl/min into an LTQ XL mass spectrometer (LTQ XL, Thermo Fisher Scientific). Mass spectra with *m*/*z* from 400 to 2000 were acquired in centroid mode. Electrospray source conditions such as ‘source fragmentation’ voltage and the tube lens voltage were optimized to help desolvation but without fragmenting the intact protein. Default values were used for most other data acquisition parameters. The resulting spectra were averaged up to 200 scans and were de-convoluted using ProMass software (Thermo Fisher Scientific).

### Analytical gel filtration

A SuperdexHR75 10/30 column (GE Health-care) equilibrated with 20 mM Tris-HCl, pH 8.0, 250 mM NaCl was calibrated with standard proteins: γ-globulin bovine (158.0 kDa), ovalbumin (44.0 kDa), CcdB (25.0 kDa), myoglobin (17.0 kDa) and vitamin B12 (1.35 kDa). Purified *Sa*MazF was run at 3.3 mg/ml in the above buffer solution. The flow rate was maintained at 0.5 ml/min, and the elution volumes and absorbance at 280 nm were recorded.

### Multi-angle light scattering

Size exclusion chromatography (SEC) coupled with multi-angle light scattering (MALS) was performed at room temperature using a Shodex packed HPLC column (Showa Denko Europe GmbH, Germany) connected to a Wyatt Technology MALS instrument. A 50 μl aliquot of protein (spinned for 30 min at 20 000 rpm in a microcentrifuge) was loaded onto the column and eluted at a flow rate of 0.2 ml/min in 20 mM Tris-HCl pH 7.0, 300 mM NaCl. The molar mass of the pure protein was calculated from the observed light scattering intensity using a refractive index (dn/dc) of 0.185 ml/g. The instrument was previously calibrated with bovine serum albumin (BSA) as standard (BSA dimer = 134 kDa and BSA monomer = 66 kDa). The results were analyzed using the ASTRA software (Wyatt Technologies, Inc.).

### Dynamic light scattering

Dynamic light scattering (DLS) data of *Sa*MazF were collected in 10 mm diameter cylindrical cuvettes at an angle of 90º employing an ALV-CGS-3 static and DLS device using a 22 mW He–Ne laser with a wavelength λ = 632.8 nm. The protein concentration of the 200 nm filtered *Sa*MazF samples was 1 mg/ml in 20 mM Tris-HCl pH 7.0, 75 mM NaCl and the range of temperature selected was from 293 K to 343 K. Measurements on *Sa*MazF were also done in the same buffer at 293 K, but with 3 M GdHCl added. Measurements on 70 nm diameter colloidal gold nano-particles (0.01 mg/ml) were used as a control to compensate for the difference in viscosity caused by the presence of GdHCl. Correlograms were recorded continuously at a fixed temperature. Data were collected in a pseudo cross-correlation setup to minimize the contribution of dead time effects and photomultiplier tube-generated artifacts after-pulsing to the recorded signal. The digital correlator outputs, from the recorded temporal dependence of the scattered intensity, the intensity autocorrelation function *g*_2_(τ)−1 with τ the delay time (56). This function *g*_2_(τ) is connected to the electric field correlation function *g*_1_(τ) through the Siegert relation

(1) \begin{equation*} g_2 (\tau ) = {B}(1 + \beta |{g}_1 (\tau )|^2 ) 
\end{equation*}

where *B* is the baseline of the correlation function at infinite delay and β the function value at zero delay. For a mono-disperse solution, *g*_1_(τ) is a single exponential decay *g*_1_(τ) = exp (−Γτ) with the decay rate Γ = *Dq*^2^ defined by the diffusion coefficient *D* of the particles and the magnitude of the scattering vector *q* = 4π*n*_0_/λ sin (θ/2) at the scattering angle θ.

DLS data were captured at fixed concentrations of *Sa*MazF at 308 K and 318 K for the total time of ∼ 4 days, at 328 K for ∼ 3 days and at 343 K for 32 h. All intensity correlation curves were fit with two exponentials.

### CD spectroscopy

Far-UV CD spectra were recorded on a J-715 spectropolarimeter (Jasco). Scans were taken using a 1 mm cuvette. Spectra of *Sa*MazF (0.2 mg/ml) were measured using different buffers in order to find the suitable buffer conditions for further experiments: 20 mM Na-phosphate pH 7.0 with 0, 75 or 300 mM NaCl, 20 mM Tris-HCl pH 7.0 with 0, 75 or 300 mM NaCl, 20 mM Na-acetate pH 5.0 and 75 mM NaCl, 20 mM Na-cacodylate pH 6.0 and 75 mM NaCl, 20 mM Na-borate pH 8.0 and 75 mM NaCl. To assess the effect of GdHCl on the structure of *Sa*MazF during the on-column separation procedure, an additional CD spectrum was recorded in 20 mM Na-phosphate pH 7.0, 75 mM NaCl, 3 M GdHCl. To minimize GdHCl absorption, a 0.2 mm cuvette was used and the *Sa*MazF concentration was 1 mg/ml. The mean residue ellipticities ([θ], degrees cm^2^ mol^−1^) were obtained from the raw data after correcting for absorption of the buffer solution according to [θ] = θ.Mw/(*N*.*c*.*l*), where Mw is the molecular weight, *c* is the mass concentration, *l* is the optical path length, and *N* is the number of amino acid residues. The temperature of the cuvette was monitored using a thermoelectric Peltier device connected with a water bath. Secondary structure predictions from CD data were performed using the CDSSTR method developed by Johnson (57,58).

### Small-angle X-ray scattering

Small-angle X-ray scattering (SAXS) data were collected in batch mode at beamline ID14-2 of the ESRF synchrotron (Grenoble, France) using a concentration series (0.5, 1.0, 3.0, 5.0 and 7.0 mg/ml) of *Sa*MazF in 20 mM Tris-HCl pH 7.0, 300 mM NaCl. The data were averaged, background-subtracted and merged to generate the scattering curve with PRIMUS (59). The radius of gyration (*R*_*g*_) was calculated from the Guinier analysis as implemented in PRIMUS and also from the entire scattering curve with the indirect Fourier Transform package GNOM (59,60). CRYSOL (61) was used to compare experimental and theoretical scattering curves. We used MODELLER (62) to model the missing residues and atoms of the ensemble consisting of all the crystal structures. The experimentally determined X-ray structures of *Sa*MazF suffice to explain to a large extent the experimental SAXS data. Therefore, the final model obtained with MODELLER (63) introduces the missing flexible C-terminus and N-terminal His-tag as well as a few missing residues in loop regions of certain monomers. The latter only results in minor structural variations in their immediate neighborhoods within the general variation seen among the different X-ray models. To define the minimal set of X-ray or NMR models that can explain the SAXS data, the minimal ensemble algorithm (Minimal Ensemble Search, MES) was used (64). This algorithm searches for the minimal ensemble set of conformations from the pool of all given conformations, systematically evaluating combinations of five models or less.

### X-ray crystallography

Crystallization conditions from Crystal Screen I and II (Hampton Research) were screened manually using the hanging drop method in 48-well plate (Hampton Research). The final successful crystallization conditions are given in Table 1. All data were collected at the PROXIMA-1 beamline of the SOLEIL synchrotron (St-Aubin, France). Data were scaled and merged using the HKL-2000 program package (65). Data collection statistics are given in Table 1. All structures were solved by molecular replacement using PHASER as implemented in the CCP4 package. For crystal form I, the coordinates of YdcE from *B. subtilis* (PDB entry 1NE8) were used as search model, while for the other crystal forms the refined coordinates of the dimer consisting of chains A and B of crystal form I were used.

**Table 1.** Crystallization, data collection and refinement

	Form I	Form II	Form III
**Crystallization**			
Protein solution	20 mg/ml in 20 mM Tris-HCl pH 7.5, 150 mM NaCl	10 mg/ml in 20 mM Na_2_HPO_4_/ NaH_2_PO_4_ pH 6.6	5.5 mg/ml in 20 mM Na_2_HPO_4_/ NaH_2_PO_4_ pH 6.6
Well solution	0.2 M NH_4_Ac, 0.1 M NaAc pH 4.6, 30% (w/v) PEG 4000	0.1 M Na-HEPES pH 7.5, 2.0 M NH_4_HCO_2_	2.0 M (NH_4_)_2_SO_4_,
			0.1 M NaAc pH 4.6
Drop contents	1 μl protein + 1 μl precipitant	1 μl protein + 1 μl precipitant	1 μl protein + 1 μl precipitant
Cryoprotection	No additional cryoprotectant added	0.075 M Na-HEPES pH 7.5, 1.4 M NH_4_HCO_2_, 30% (v/v) glycerol	1.6 M (NH_4_)_2_SO_4_,
			0.08 M NaAc pH 4.6, 20% (v/v) glycerol
**Data collection**			
Resolution range (Å)	56.34–2.10 (2.16−2.10)	46.0–2.3 (2.53–2.30)	39.2–2.7 (2.78–2.70)
Space group	P2_1_2_1_2_1_	C222_1_	C222_1_
Unit cell (Å)	*a* = 60.72	*a* = 72.58	*a* = 90.88
	*b* = 65.36	*b* = 92.00	*b* = 92.63
	*c* = 112.01	*c* = 71.52	*c* = 222.37
Mosaicity (°)	0.21–0.61	0.36–0.58	0.69–0.88
Total no. of measured intensities	144 316	210 742	87 263
Unique reflections	26 598 (2150)	10 915 (1930)	25 526 (2057)
Multiplicity	5.4 (4.2)	5.7 (5.8)	3.4 (3.4)
Mean I/*σ*(I)	14.0 (3.4)	11.2 (3.5)	13.3 (2.9)
Completeness (%)	99.8 (98.8)	99.8 (99.9)	98.0 (97.7)
*R*_sym_	0.104 (0.341)	0.137 (0.734)	0.093 (0.406)
Wilson B factor (Å^2^)	30.3	26.4	53.2
Solvent content (%)	34	38	36
**Refinement**			
*R*_cryst_	0.175 (0.163)	0.195 (0.220)	0.208 (0.274)
*R*_free_	0.228 (0.237)	0.248 (0.319)	0.242 (0.326)
Most favored regions (%)	98.1	98.2	94.7
Allowed regions (%)	1.9	1.8	4.6
Disallowed regions (%)	0.0	0.0	0.7
RMSD bond lengths (Å)	0.013	0.009	0.014
RMSD bond angles (º)	1.18	1.29	1.33
Content of the asymmetric unit	Two dimers	One dimer	Four dimers
Average B-factor of all atoms (Å^2^)	36.6	35	46.2
Average B-factor of solvent atom (Å^2^)	39.9	38.4	-
No. of protein atoms	3615	1743	6712
Total no. of missing residues	86	39	146
No. of missing residues/chain (N-term, loop, C-term)			
Monomer A	12, 0, 6	12, 0, 7	13, 0, 6
Monomer B	12, 5, 7	13, 0, 7	12, 0, 7
Monomer C	12, 6, 7	-	13, 0, 7
Monomer D	12, 0, 7	-	13, 0, 7
Monomer E	-	-	13, 2, 7
Monomer F	-	-	13, 0, 6
Monomer G	-	-	13, 0, 7
Monomer H	-	-	13, 0, 6
No. of water molecules	218	69	0
PDB entry	4MZM	4MZT	4MZP

All structures were refined against a maximum likelihood target using Phenix (66). After initial rigid body refinement, a Cartesian simulated annealing protocol (starting at a Boltzmann temperature of 5000 K) was performed to uncouple R-work and R-free. This was followed by rounds of positional and isotropic B-factor refinements interspersed by manual rebuilding using Coot (67). At the end of the refinement, waters were included in the model where relevant, and translation-libration-screw (TLS) parameters (one TLS group per chain) were included in the refinement. For crystal forms I and II, non-crystallographic symmetry (NCS) restraints were applied at the start of the refinement and released based on monitoring R-free. For crystal form III, NCS restraints were maintained throughout the refinement except for loops Gly48-Lys54 and Ile61-Lys70. The final refinement statistics are given in Table 1.

In all structures, most of the residues constituting the N-terminal His-tag are disordered and the model starts at Pro1, except for all chains in form I and chain A in form II where it starts at Asp0, and chain B of form III, which starts at Gln-1. At the C-terminus, most chains end at Asn113 except for chain A of form I and chains A, F and H of form III that end at Ala114. In addition, electron density is missing for residues Ile50-Lys52 (form II chain B), Arg49-Lys52 (form II chain C) and Lys63-Lys65 (form II chain E).

### Analysis of crystal packing contacts

For each space group, all MazF-MazF contact interfaces within the unit cell were generated and evaluated using the PDBePISA webserver (68). The database of crystal packing contacts generated therefrom was grouped per chain, screened for redundancy and truncated to unique contacts only. The per residue buried surface area was used as a metric to gauge the involvement of individual residues in the symmetry mates interface. For each chain, values of buried surface area were summed per residue for all the interfaces and plotted as a function of primary sequence.

### NMR structure determination

^13^C- and ^15^N-labeled *Sa*MazF was prepared at 1 mM in 20 mM Na phosphate pH 6.6, 10% D_2_O. All NMR spectra were recorded at 308 K using a Varian NMR Direct-Drive Systems 800 MHz spectrometer equipped with a salt tolerant triple-resonance PFG-Z cold probe. Two-dimensional NOESY and three-dimensional ^15^N and ^13^C NOESY-HSQC spectra with 100 ms mixing times were recorded on the same sample. All NMR data were processed using NMRPipe (69) and analyzed by CCPNMR (70) or NMRView (71). The assignment of backbone and side-chain ^1^H, ^15^N and ^13^C resonances were described previously (54).

Twenty inter-monomeric nuclear Overhauser effects (NOEs) were identified based on a preliminary model of the *Sa*MazF calculated from chemical shifts using the CS-Rosetta software (72) and the dimeric structure of YdcE (PDB entry 1NE8), the closest homolog of *Sa*MazF present in the Protein Data Bank. These manually assigned NOEs were used together with non-assigned NOEs and dihedral restraints from Talos+ (73) as input for the structure calculations using CYANA version 2.1. Non-assigned NOEs were assigned using the automated NOE assignment procedure of CYANA (74,75). A standard protocol was used with seven cycles of combined automated NOE assignment and structure calculation of 100 conformers in each cycle. From the three NOESY data sets, 3262 NOEs were unambiguously assigned, including 66 inter-monomeric NOEs (Table 2). These unambiguously assigned restraints were used for a final structure refinement in explicit solvent using the RECOORD protocol (76), which runs under CNS (77). The twenty lowest-energy structures were used for final analysis.

**Table 2.** NMR structure determination

**NMR structural statistic**	
**Distance restraints**	
Short range (*i* − *j* = 0)	826
Medium range (1 ≤ |*i* − *j*| ≤ 4)	1358
Long range (|*i* − *j*| ≥ 5)	1012
Inter monomer (A to B)	66
Total	3262
**Dihedral restraints**	
Phi angles	77
Psi angles	74
**Restraint statistics**	
NOE violations > 0.5 Å	2.25 ± 1.74
Dihedral violations > 5º	2.4 ± 2.4
**RMSD (a.a. 1–47, 56–112) from average (Å)^a^**	
Backbone N, CA, C', O	0.65 ± 0.09
Heavy atoms	1.03 ± 0.15
**Ramachandran plot**	
Most favored regions (%)	85.7
Additional allowed regions (%)	13.6
Generously allowed regions (%)	0.6
Disallowed regions (%)	0.0
PDB entry	2MF2

^a^Flexible N- and C-terminal residues and residues of loops 48–55 and 63–70 were omitted from the RMSD analysis and Ramachandran statistics obtained from PROCHECK analysis.

### Backbone dynamics from ^15^N relaxation data

The relaxation parameters ^15^N R1, R2, and ^1^H–^15^N steady-state NOEs were measured at 599.78 MHz and 308 K. Relaxation values were obtained from series of 2D experiments with coherence selection achieved by pulse field gradients using the experiments described previously (78) on ^13^C^15^N-labeled *Sa*MazF. The ^1^H–^15^N heteronuclear NOEs were determined from the ratio of peak intensities (*I*_on_/*I*_off_) with and without the saturation of the amide protons for 3 s. Average heteronuclear NOE values and their errors were obtained from a duplicate set of experiments. ^15^N R1 and ^15^N R2 relaxation rates were measured from spectra with different relaxation delays: 100, 200, 300, 400, 500, 600, 700, 900, 1200 and 1500 ms for R1 and 10, 30, 50, 70, 90, 110, 130, 150, 170 and 210 ms for R2. Relaxation parameters and their corresponding errors were extracted with the program NMRView (71). Estimation of the rotational correlation time of *Sa*MazF from the ^15^N R2/R1 ratio was done using TENSOR2 (79).

### Chemical shift mapping

NMR titrations were recorded at 308 K on a Varian NMR Direct-Drive Systems 800 MHz spectrometer equipped with a salt tolerant triple-resonance PFG-Z cold probe. A *Sa*MazE-derived C-terminal peptide (residues 23–56 obtained as lyophilized powder from Bio-Synthesis, Lewisville, TX, USA; *Sa*MazE^23–56^) was re-suspended in 20 mM phosphate pH 6.6 at a concentration of 3.5 mM and titrated into a solution of 0.5 mM ^13^C^15^N-labeled *Sa*MazF in the same buffer in eight steps to a final molar ratio of *Sa*MazF_2_:*Sa*MazE^23–56^ of 1:2. A ^15^N-HSQC of the *Sa*MazF in absence of the *Sa*MazE-derived peptide was recorded as reference. ^15^N-HSQC spectra were further recorded after each addition of *Sa*MazE^23–56^. The magnitude of the chemical shift perturbation (Δδ) was calculated by

(2) \begin{equation*} \Delta \delta = [(\Delta \delta _{\rm H} )^2 + (\Delta \delta _{\rm N} /6.51)^2 ]^{1/2}, 
\end{equation*}

where Δδ is the difference between the bound and free form combined chemical shifts.

### Residue conservation

Residue conservation scores were calculated using ConSurf (80) based either on the 12 pre-aligned sequences in Supplementary Figure S1 or based upon a Clustal W (81) multiple sequence alignment of 19 randomly selected MazF sequences with sequence identities with *Sa*MazF exceeding 35% (uniprot entries MAZF_STAHJ, ENDOA_BACSU, R9KFQ5_9FIRM, B2GA66_LACF, R5L321_9CLOT, E7G757_9FIRM, F0SUX5_SYNGF, E6UK99_RUMA7, E3EKA9_PAEPS, F7V2M0_CLOSS, F8HY64_WEIKK, K9W9N9_9CYAN, K2B658_9BACT, B1BUR9_CLOPF, D8FFQ1_9DELT, N9YMI4_9CLOT, I0XX63_9LEPT, R5NJH8_9FIRM and F3AL12_9FIRM) next to the *Sa*MazF sequence itself.

### Modeling of the *Sa*MazF-RNA complex

We used the structure of the *Bacillus subtilis* MazF (YdcE) in complex with RNA (PDB entry 4MDX) as template for building a model of the *Sa*MazF-RNA complex. The conformation of the loop comprised of residues 48–58 of *Sa*MazF (crystal form I) was rebuilt using the program MODELLER (62) to generate the RNA-bound conformation observed in YdcE. The RNA from PDB entry 4MDX was transferred to this model of *Sa*MazF in its RNA-binding conformation by superposition with PDB entry 4MDX. The resulting *Sa*MazF-RNA complex was then relaxed in two minimization steps, using the program NAMD (82), first in vacuum and subsequently in an explicit water environment (4605 TIP3 water molecules in a sphere with radius 35 Å around the centre of mass of the *Sa*MazF dimer).

## RESULTS

### Purification of *Sa*MazF

**Figure 1.**
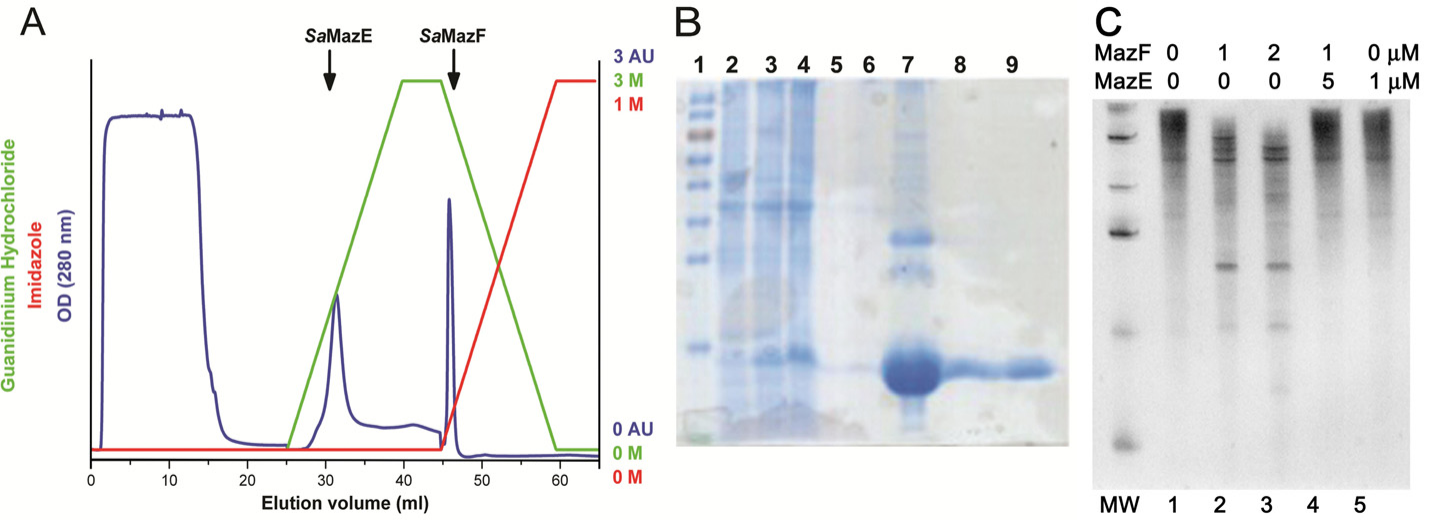
Purification of *Sa*MazF. (**A**) Ni-NTA purification of *Sa*MazE and *Sa*MazF. After loading, *Sa*MazE is eluted using a gradient of guanidinium hydrochloride while *Sa*MazF remains bound to the column. *Sa*MazF is eluted using an immidazole gradient and subsequently dialyzed to remove the guanidinium. (**B**) SDS-PAGE showing the progress of expression and purification. Lane 1: molecular weight marker (Fermentas PageRuler). Lane 2: *E. coli* extract prior to induction. Lane 3: *E. coli* 2 h post induction. Lane 4: *E. coli* extract after overnight induction. Lane 5: *Sa*MazE eluted from the Ni-NTA column. Lane 6: fractions in between the *Sa*MazE and *Sa*MazF peaks. Lane 7: *Sa*MazF eluted from the Ni-NTA column. Lanes 8 and 9: *Sa*MazF after further purification on SEC. (**C**) RNase activity of *Sa*MazF. The figure shows the ribonuclease activity of *Sa*MazF against bacteriophage MS2 genomic RNA. Lane 1: New England Biolabs Inc. low range ssRNA ladder (50, 80, 150, 300, 500 and 1000 bases). Lane 2: intact RNA control, excluding any nonspecific RNase contamination. Lanes 3 and 4: cleaved RNA by an active *Sa*MazF at 1 μM and 2 μM, respectively. Lane 5: RNA degradation inhibition of *Sa*MazF by the presence of *Sa*MazE. Lane 6: *Sa*MazE sample incubated with RNA.

*Sa*MazF is lethal to *E. coli* when over-expressed and can only be obtained if co-expressed with its antitoxin *Sa*MazE. Therefore, the sa*mazE* and sa*mazF* genes were introduced in the pETDuet1 (Novagen) expression vector, which attaches a histidine-tag to the N-terminus of *Sa*MazF. Upon induction with 1 mM IPTG, this leads to considerable production of *Sa*MazF without compromising cell viability. To obtain pure and well-folded *Sa*MazF, a purification method was devised that allows removal of non-covalently bound *Sa*MazE without disrupting the correct folding of *Sa*MazF (Figure 1A and B). First, a Ni-NTA column is used to trap *Sa*MazE–*Sa*MazF complexes and the column is extensively washed to remove all contaminants. To remove *Sa*MazE, a gradient of guanidinium hydrochloride (GdHCl) is used, which disrupts the *Sa*MazE–*Sa*MazF interaction. Here it is crucial to reduce the time of the GdHCl treatment as well as the maximal concentration used as the resulting *Sa*MazF otherwise irreversibly aggregates. Likely, under the conditions used, *Sa*MazF retains its dimeric state on the column (see below) and we assume that this is key for obtaining a sample of well-folded *Sa*MazF. While the concentration of GdHCl on the column is reduced, the protein is eluted using a gradient of imidazole. The protein elutes at about 125 mM imidazole and 2.75 M GdHCl, after which it is dialyzed to remove both these components. A final gel filtration step on a Superdex 75PG column removes any further contaminants. This method allowed producing significant amounts of pure *Sa*MazF (25–35 mg from 1 l of culture).

To exclude the possibility that either the GdHCl treatment or the presence of the N-terminal His-tag might hamper the functionality of *Sa*MazF, we evaluated its *in vivo* and *in vitro* activities. Non-tagged as well as N-terminal and C-terminal His-tagged *SamazF* constructs prevent colony formation upon induction of the Plac promoter with IPTG, but not when repressed by glucose (data not shown). The ribonuclease activity of the purified protein was assayed using the 3569 nucleotide genomic RNA of bacteriophage MS2 (83). As shown in Figure 1C, we find *Sa*MazF to be able to cleave MS2 RNA. Furthermore, this activity is inhibited by the presence of the antitoxin *Sa*MazE. The latter indicates that the RNase activity results from *Sa*MazF and not from any other contaminating ribonuclease.

### Biophysical and biochemical properties of *Sa*MazF

**Figure 2.**
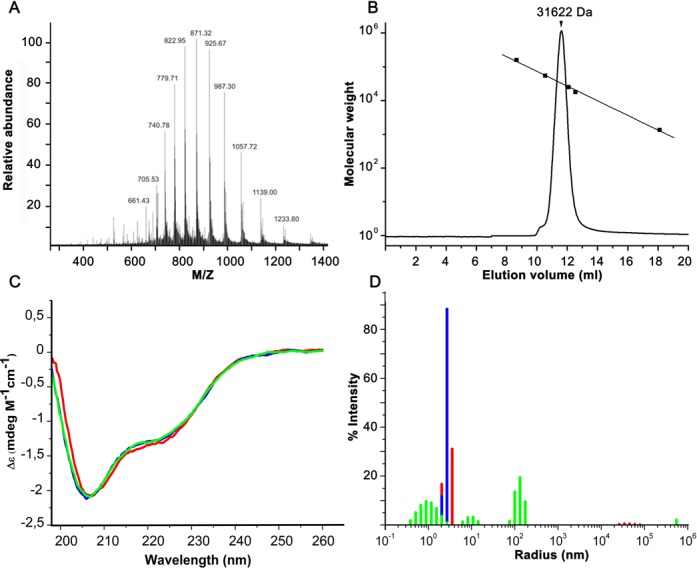
Biophysical characterization. (**A**) Electrospray mass spectrum of *Sa*MazF. The *m*/*z* values for the major peaks are indicated. (**B**) Analytical gel filtration. Shown is the elution profile of *Sa*MazF on a superdex HR75 10/30 column together with the elution volumes of four molecular weight standards (bovine gamma-globulin, 158.0 kDa; ovalbumin, 44.0 kDa; myoglobin–F-plasmid CcdB, 25.4 kDa and 17.0 kDa; vitamin B12, 1.35 kDa) plotted versus their molecular weights. (**C**) CD spectra of *Sa*MazF in 20 mM Na-phosphate pH 7.0 and at different concentrations of NaCl (0 mM green, 75 mM blue and 300 mM red). (**D**) DLS-derived intensity versus radius histogram of *Sa*MazF under the same conditions as in panel (B). The same color scheme is used.

The resulting protein shows a single band on SDS-PAGE, and its identity was confirmed by electrospray mass spectrometry (Figure 2A). The derived mass of 14 794 ± 2.4 Da is in close agreement with the theoretical mass of 14 791.9 Da for the *Sa*MazF monomer lacking its N-terminal methionine but including the N-terminal His-tag (GSSHHHHHHSQDP). The protein elutes with an apparent molecular weight of about 31 500 Da in an analytical gel filtration experiment indicating a homodimer (Figure 2B). *Sa*MazF shows CD spectra reminiscent of a folded protein in different buffer and salt conditions (Figure 2C and Supplementary Figure S2A and B). CD spectra of *Sa*MazF at 293 K under a range of conditions show a pronounced minimum at 208 nm and a weaker minimum at 222 nm. Analysis of the CD spectra using CDSSTR indicates the presence of 10% α-helix and 25% β-sheet, which compares reasonably well with the values of 15% and 28%, respectively, calculated from the crystal and NMR structures (see below). In addition, the quality of the protein is such that crystals can be obtained and good-quality NMR spectra can be collected from ^13^C^15^N-labeled material (53,54).

The oligomeric state of *Sa*MazF was further investigated using MALS (determined MW: 30.7 kDa) and DLS. DLS experiments show that *Sa*MazF aggregates at very low ionic strengths in absence of salt, but that an essentially mono-disperse sample is obtained at low (75 mM NaCl, 20 mM Tris-HCl pH 7.0) and high (300 mM NaCl, 20 mM Tris-HCl pH 7.0) salt concentrations (Figure 2D). The derived hydrodynamic radius and corresponding calculated molecular weight are 2.6 nm and 32 kDa for the low salt condition and 2.5 nm and 29 kDa for the high salt condition, respectively, in agreement with a well-structured *Sa*MazF dimer.

### Thermal unfolding of *Sa*MazF

**Figure 3.**
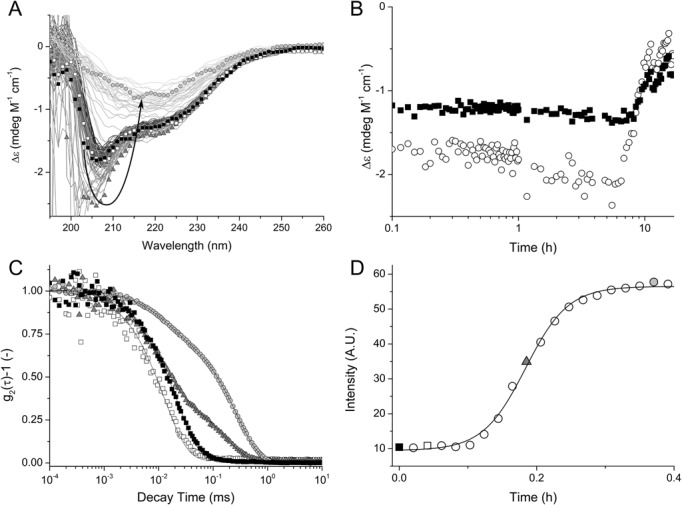
Thermal stability of *Sa*MazF. (**A**) CD spectra of *Sa*MazF at 293 K (white squares) and at different time intervals at 343 K (thin lines). Spectra corresponding to key structural states are indicated by symbols (*t* = 0 min, open circles; *t* = 270 min, gray triangles; *t* = 960 min, gray circles). The initial CD signals at 293 K and 343 K are essentially identical. After a lag phase, the minimum at 207 nm deepens, followed by a slow transition to a mainly β-structure containing state. (**B**) CD signal at 207 nm (white circles) and 220 nm (black squares) at 343 K followed in function of time. The duration of the lag phase is strongly dependent on temperature and protein concentration, indicating a nucleation event. (**C**) Normalized intensity correlation functions of a 0.2 μm filtered buffered *Sa*MazF solution (20 mM Tris-HCl pH 7.0, 75 mM NaCl) after 0 min of incubation at 343 K (black squares), 2.5 min (open squares), 11 min (gray triangles) and 22 min (gray circles), respectively. Full lines represent fits with Equation (1). At *t* = 0, the correlation function is well characterized by a single exponential decay with a characteristic time of 2.5 ± 0.1 × 10^−2^ ms, indicative of the monodisperse nature of the sample. After 7 min of incubation at 343 K, a second decay appears in the correlation function, which is correlated with an intensity increase of the scattered light. This corresponds to the formation of a second, ‘slower’ species in solution, considerably larger than a native MazF dimer. Both the relative amplitude and the decay time of the second population increase as a function of incubation time, corresponding to an increase in characteristic size and number density, e.g. 36 ± 5 nm for *t* = 11 min and 49 ± 5 nm for *t* = 22 min. Conversely, the characteristic size of the ‘faster’ species (presumed native *Sa*MazF dimer) is constant as a function of time suggesting that the overall fold is unperturbed, i.e. 2.7 ± 0.2 nm, 2.8 ± 0.3 nm, 2.6 ± 0.3 nm and 2.7 ± 0.2 nm for *t* = 0, 2.5, 11 and 22 min, respectively. (**D**) Scattered intensity at 343 K as a function of time: full line represents a Boltzmann sigmoidal curve fit. The data points indicated as grey triangle or black and open square correspond to the equivalent curves in panel C.

When attempting to obtain data on the thermal stability of *Sa*MazF, we observed that the CD spectrum of *Sa*MazF measured within minutes of heating the protein to 371 K only shows minor differences with the corresponding CD spectrum at 293 K (Supplementary Figure S2C). To distinguish between a very high thermal stability with a melting temperature above 371 K and a high kinetic barrier for thermal unfolding, we followed the CD signal at different temperatures as a function of time (Figure 3A and B). These experiments show a temperature-dependent lag phase followed by two apparent structural transitions for temperatures of 328 K and above. At lower temperatures (318 K and below), the CD spectra remain constant for at least one week. The first structural transition is characterized by a deepening of the CD minimum around 207 nm (Figure 3A). Analysis of these spectra indicates that the β-sheet content is reduced and that helix content (most likely polyproline II) increases. This is followed by a second structural transition toward a species with a high (45%) β-sheet and lacking α-helix.

The previous observations suggest a nucleation process preceding aggregation. This was examined by DLS measurements (Figure 3C and D) that show a starting state of particles with a hydrodynamic radius of 2.6 nm, in agreement with the size of the *Sa*MazF dimer determined by X-ray crystallography and NMR spectroscopy. In time, a considerably larger second species develops, again after a temperature-dependent lag time. This aggregation process masks any unfolding event, and the discrimination between thermodynamic and kinetic stability of *Sa*MazF cannot be based on these data alone. Nevertheless, as the aggregation involves a significant structural transition, it seems likely that kinetically determined unfolding creates the starting point from which aggregation nuclei can grow.

### Crystal structures of *Sa*MazF

**Figure 4.**
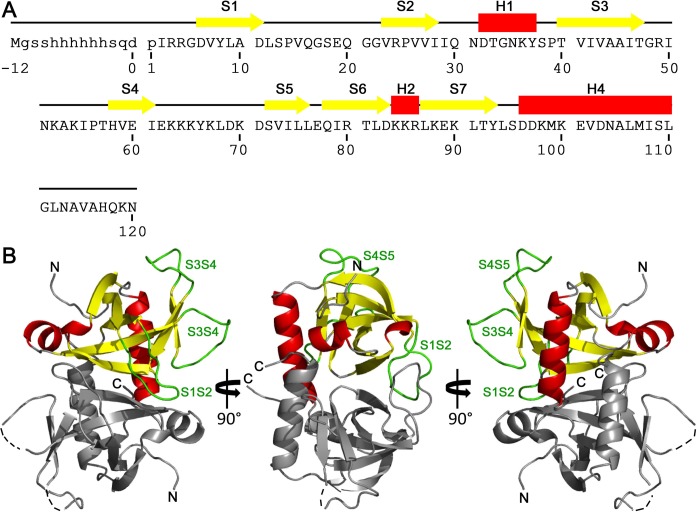
Overall structure of *Sa*MazF. (**A**) Amino acid sequence of *Sa*MazF. Secondary structure elements derived from the X-ray structures of *Sa*MazF are indicated by yellow arrows (β-strands) and red bars (α-helices) and are labeled. (**B**) Overall structure of the *Sa*MazF dimer. Shown is a cartoon figure of the dimer formed by chains A and B of crystal form I. Chain A is colored according to secondary structure as in (A). Loop regions Leu12-Gly22, Gly48-Lys54 and Lys64-Lys70 are colored green and labeled as S1-S2, S3-S4 and S4-S5, respectively. Chain B is shown in gray. N- and C-termini are indicated. Dotted lines show the connection between the extremities of loops that lack electron density. Panel (B) was prepared using PyMol (84).

**Figure 5.**
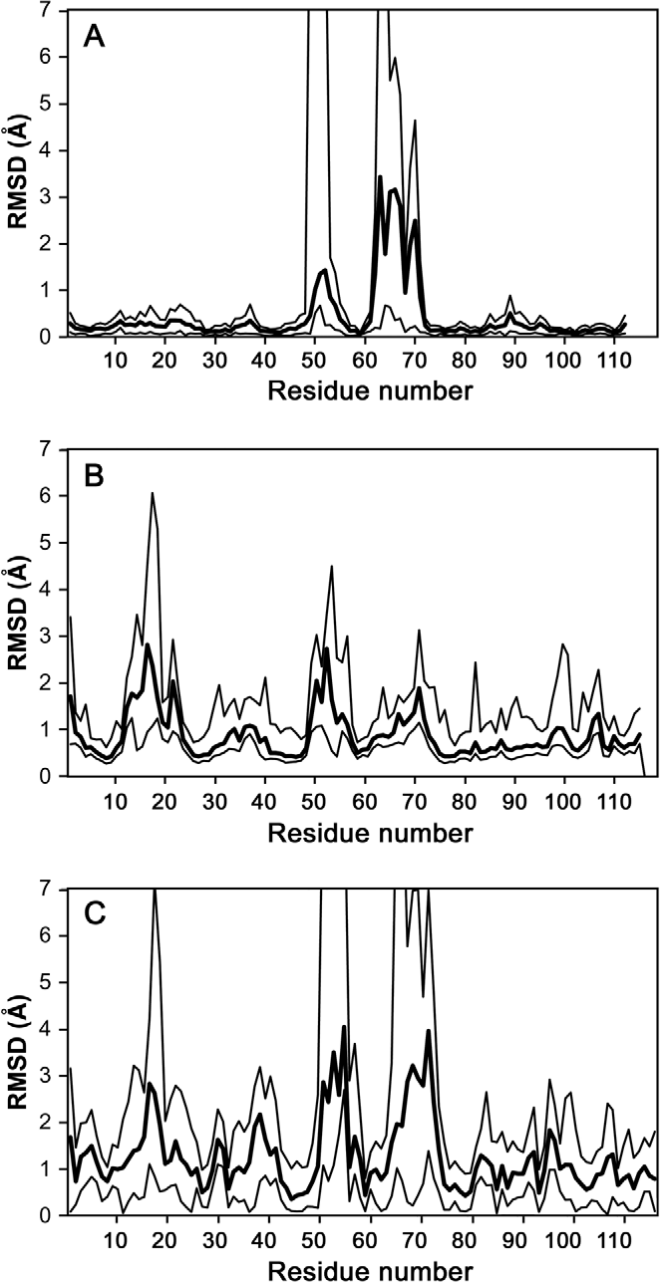
Structural variability of *Sa*MazF. (**A**) Per residue RMSDs within the X-ray ensemble. The mean RMSDs for all pair-wise comparisons of *Sa*MazF monomers within the X-ray ensemble (seven independent monomers—form III is represented by a single monomer only because of the imposed NCS restraints) are shown as a bold line. The minimum and maximum values for each residue are represented by the thin lines. When no coordinates were available (due to lack of electron density), an arbitrary RMSD of 10 Å was used. The largest variability is seen for amino acids Lys64-Lys70 and to a lesser extent for Gly48-Lys54. (**B**) Per residue RMSDs within the NMR ensemble. Similar plot as in (A), but now using the 20 lowest energy NMR structures that were deposited in the Protein Data Bank. The largest variability is seen for amino acids Leu12-Ser18, Gly48-Lys54 and Lys64-Lys70. (**C**) Comparison of the X-ray and NMR ensemble. Plotted are the mean RMSDs for all pair-wise comparisons of *Sa*MazF monomers in the X-ray ensemble with those in the NMR ensemble.

Three different crystal forms of *Sa*MazF are available (Table 1), which lead to the structures of 14 crystallographically independent *Sa*MazF monomers forming 7 independent dimers (Table 1). Each of these monomers was independently refined except for the eight monomers present in crystal form III, which were restrained by NCS because of the lower resolution (excluding two more variable loops that clearly adopt distinct conformations). Figure 4 shows the overall structure of *Sa*MazF. *Sa*MazF adopts the typical MazF/CcdB fold consisting of a 5-stranded anti-parallel β-sheet (strands S1–S3 and S6–S7) followed by a 4-turn α-helix (H3 and further decorated with a small 3-stranded anti-parallel β-sheet (strands S3–S5 with S3 taking part in both sheets), a short 2-turn α-helix (H1) and a 1-turn helix H2 (see Figure 4 for definitions). Overall, the structures of the *Sa*MazF monomers are very similar (Figure 5A and Supplementary Figure S4) with pair-wise backbone root-mean-square deviations (RMSDs) of 0.18–0.58 Å for all 99 residues defined in each molecule (the 8 NCS restrained monomers from crystal form III are represented in this comparison by chain A only). Structural variation is seen at the N- and C-termini and in two loop regions: Gly48-Lys54 (between strands S3-S4) and Ile61-Lys70 (between strands S4-S5). In some monomers, parts of these loops lack electron density and are, together with differences in N- and C-termini, responsible for the different number of residues found in the different X-ray structures. The conformations observed for loop S3-S4 can be considered to belong to a single family, but in loop S4-S5 highly distinct conformations are observed that are related to crystal packing (see below).

The *Sa*MazF dimer is formed by pairing strand S6 from two monomers to form a dimer-wide 10-strand anti-parallel β-sheet. Further contacts include the anti-parallel alignment of the last turn of helix H3 and an extensive series of hydrophobic side-chain to side-chain contacts involving residues Ile29, Ile42, Ile79, Leu106 and Ile110 that create an extended hydrophobic core crossing the dimer interface. Superposition of all seven *Sa*MazF dimers show that the dimer is highly rigid (Supplementary Figure S4), with no significant inter-monomer rotation being detected.

### Crystal packing

**Figure 6.**
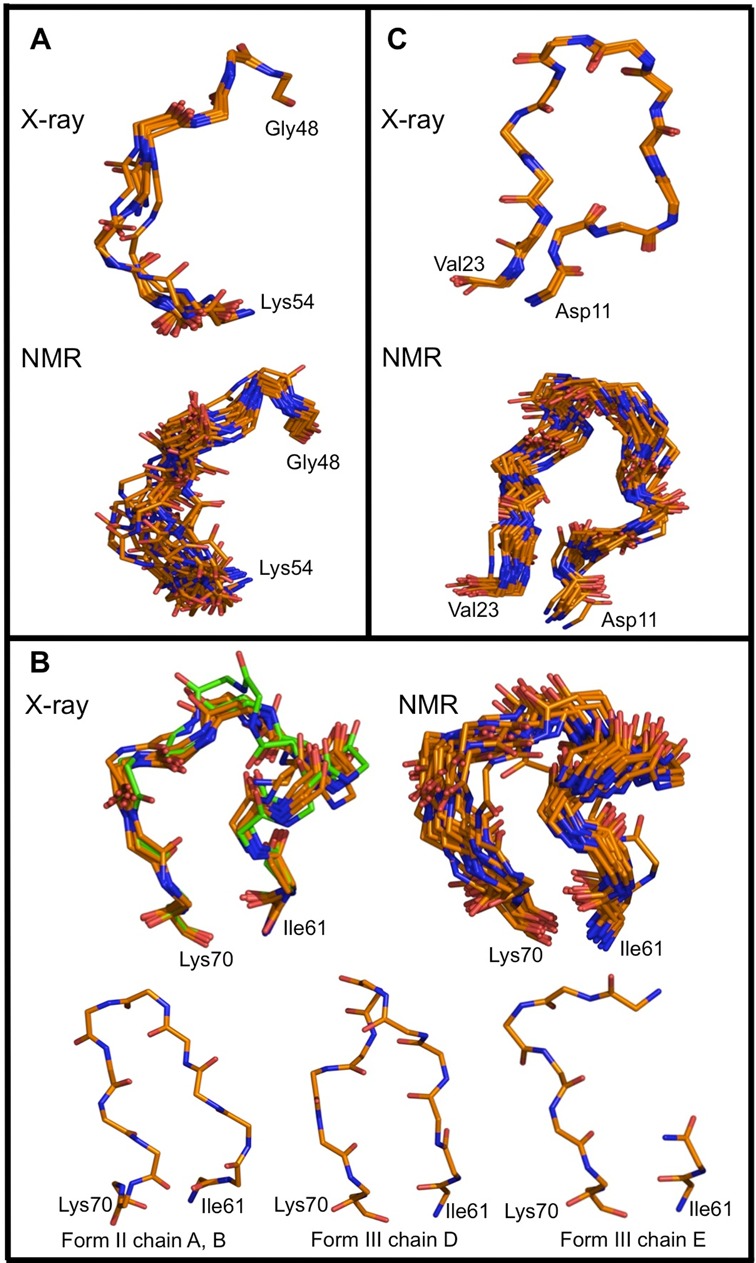
Crystal packing. (**A**) Stick representation of the backbone conformations of loop Gly48-Lys54 in the X-ray ensemble (above) and in the NMR ensemble (below) and colored according to atom type (carbon, orange; nitrogen, blue; oxygen, red). Within the X-ray ensemble, this loop is involved in crystal packing in each chain. (**B**) Stick representation of the backbone conformations of loop Ile61-Lys70. The ‘canonical’ conformation observed in the crystal structures in 10 out of 14 chains is shown in the upper left of the panel. Colored as in (A) except for the two chains that are not in packing contacts where carbons are drawn in green. The equivalent NMR ensemble is shown in the upper right of the panel while the three packing-driven conformations are shown at the bottom of the panel. (**C**) Stick representation of the backbone conformations of loop Leu12-Ser18 in the X-ray ensemble (above) and in the NMR ensemble (below). Coloring as in (A). This figure was prepared using PyMol (84).

As the solvent content of all three crystal forms is very low, it is not unlikely that lattice contacts influence the conformation of the protein. Supplementary Figure S3 plots the amount of surface area buried in crystal lattice contacts for each chain in function of residue number. From these plots, it can be seen that lattice contacts are not randomly distributed on the protein surface. In particular, among the two loops that show higher RMSD values in the X-ray ensemble, loop S3-S4 (Gly48-Lys54) is involved in lattice contacts in all structures (Figure 6A). It is unlikely, however, that crystal lattice interactions have a major influence on the conformation of this rather extended loop given that all conformations observed seem to belong to a single family, with only two individual conformations (form I chain D and form II chain A) deviating somewhat from the canonical conformation. In the absence of a chain where this loop is not involved in lattice interactions, it nevertheless remains difficult to draw hard conclusions.

Loop S4-S5 (Ile61-Lys70) is involved in lattice contacts in most but not all *Sa*MazF monomers. Four classes of conformations are observed (Figure 6B). The most common conformation is observed in ten chains, two cases of which do not involve lattice contacts. In the remaining four chains, this conformation is prohibited as it would lead to steric clashes with a neighboring monomer. Of these remaining chains, form II chains A and B adopt the same conformation while form III chains D and E each adopt a unique conformation. Loops S4-S5 of the latter four chains are all involved in lattice contacts. Thus, it seems like loop S4-S5 will adopt a default conformation when the crystal environment allows for it, but will adapt its conformation otherwise.

Finally, loop S1-S2 (Leu12-Ser18) adopts the same conformation in all monomers independent of its involvement in the crystal environment (Figure 6C). This loop does, however, show a high RMSD in the NMR ensemble (see below).

### NMR solution structure

The solution structure of *Sa*MazF was obtained using a combination of unambiguous automatically assigned NOEs in CYANA, additional manually assigned NOEs and dihedral angle restraints obtained from Talos+ analysis in a water-refinement protocol using RECOORD. The resulting ensemble of the 20 lowest energy structures (Supplementary Figure S4) shows very good Ramachandran statistics while fulfilling the experimental data (Table 2). Pair-wise backbone RMSDs of these 20 monomers range from 0.59 Å to 1.20 Å (Figure 5B). The NMR-derived secondary structure elements correspond to those identified in the X-ray structures, and structural variability is limited to loop regions Leu12-Ser18 (S1-S2), Gly48-Lys54 (S3-S4) and Lys64-Lys70 (S4-S5), as well as the N- and C-termini.

Although the NMR ensemble agrees well with the ensemble of X-ray-derived structures, they cannot be considered identical (Figure 5C and Supplementary Figure S4). The pair-wise RMSDs between NMR and X-ray structures vary between 1.02 Å and 1.58 Å, higher than the internal variation within the NMR and X-ray ensembles. This suggests that the X-ray ensemble, while less divergent than the NMR ensemble, is not a simple subset of the NMR ensemble and that the larger structural diversity of the NMR ensemble compared to the X-ray ensemble cannot be attributed solely to the lower accuracy of NMR structures (due to the smaller data-to-parameter ratio). Thus, lattice interactions seem to affect the X-ray structures even if averaged out over several crystal environments.

Analysis of the pair-wise RMSD plots of both the NMR and the X-ray ensemble shows that differences between the NMR and X-ray ensembles are spread out over the whole sequence, but are maximal in those regions where the NMR and X-ray ensembles also differ most within each ensemble. In those regions, the NMR models vary much more than the X-ray models. Most noticeable is the loop region Leu12-Ser18 (S1-S2), which adopts essentially one single conformation within the X-ray ensemble but is highly variable within the NMR ensemble. Also, region Thr33-Thr40 including helix H1 seems to contribute to the systematic differences between both ensembles and shows a smaller peak in structural variability within the NMR ensemble.

**Figure 7.**
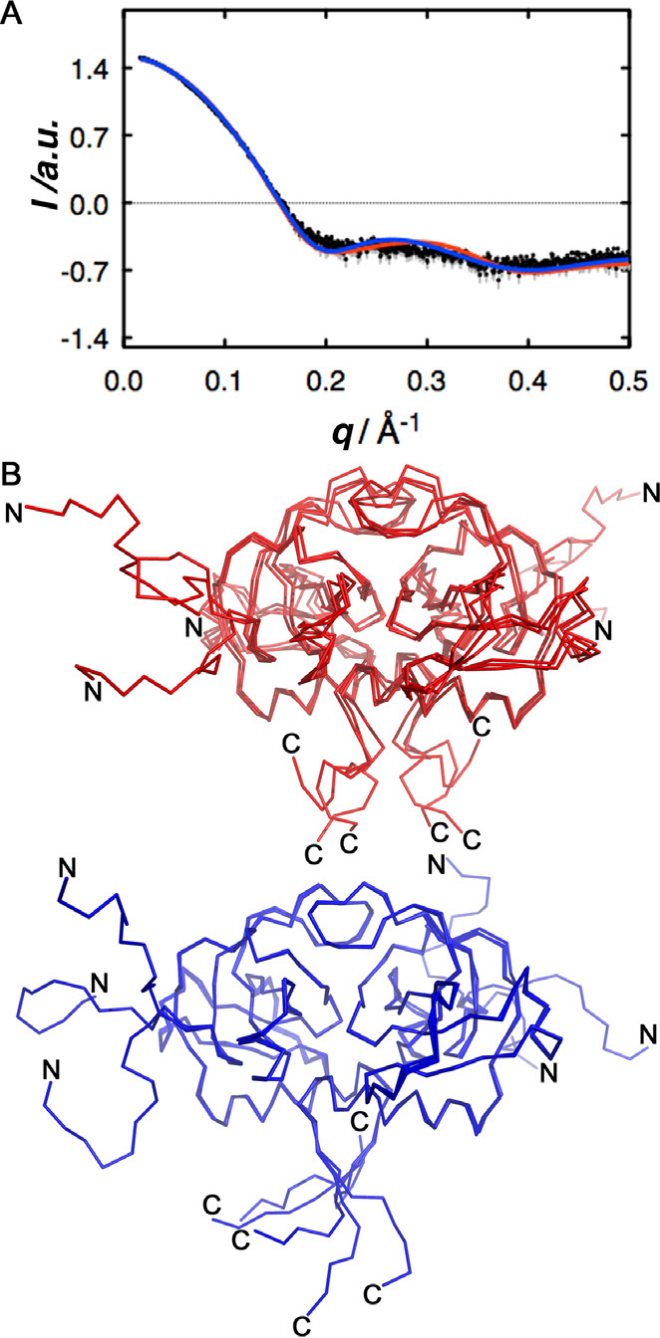
Small-angle X-ray scatter. (**A**) Experimental scatter data. The experimental data are shown in black while the error margins are shown in gray. Analysis of the scattering curve indicates that *Sa*MazF forms a globular dimer with a radius of gyration of 23.1 Å as determined through Guinier and *p*(*r*) analysis, and a molecular weight of about 28 kDa as determined through Guinier analysis. The theoretical scattering curves calculated from the full NMR (red) and X-ray (blue) ensembles are overlaid and predict the experimental data equally well. (**B**) Minimal set of NMR (red) and X-ray (blue) structures necessary to predict the experimental data. In each case, selecting three models from the full ensemble is sufficient, with the major source of variability that needs to be taken into account coming from the disordered C-terminus and the N-terminal His-tag (indicated by N and C). Panel (B) was prepared using PyMol (84).

Both the NMR and X-ray ensembles were further validated by comparing how well they are able to predict the experimentally measured SAXS data (Figure 7). Table 3 shows all the structural parameters derived from the Guinier analysis. After modeling the N- and C-termini, missing loops and missing atoms in the X-ray ensemble, both ensembles fit the experimental SAXS data quite well (Table 3). We looked for the minimal ensemble sufficient to describe the SAXS data, which in both cases turned out to be as little as three models. The major source of variability that is required for a good agreement with the SAXS data is found at the flexible C-terminus and the N-terminal His-tag (Figure 7B).

**Table 3.** Structural parameter determined from the Guinier analysis of the experimental SAXS curve of *Sa*MazF

Protein	*R_g_* (Å)	*D*_max_ (Å)	MW (kDa) SAXS	MW (kDa) theoretical	*χ* NMR	*χ* X-ray
*Sa*MazF	23.09	79.8	28.3	29.8	1.06	1.17

### Conformational flexibility and backbone dynamics from ^15^N relaxation data

**Figure 8.**
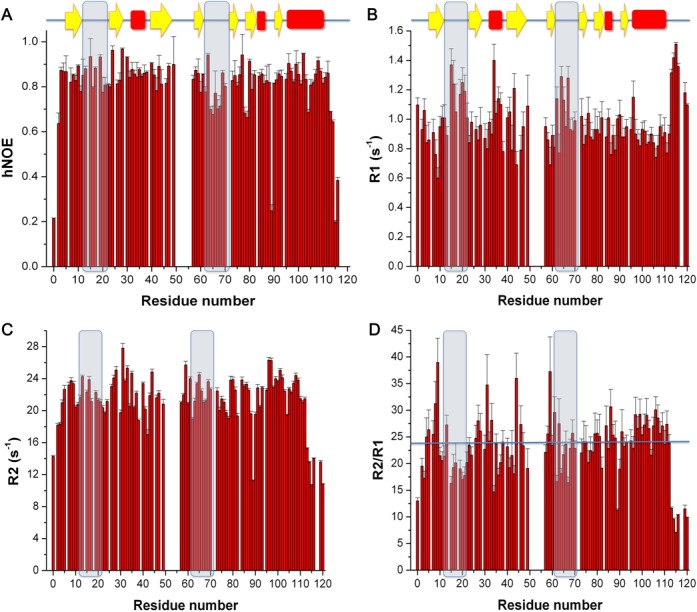
Backbone dynamics of *Sa*MazF. Backbone dynamics of *Sa*MazF were measured at 600 MHz and 308 K. (**A**) ^1^H–^15^N steady-state heteronuclear NOEs in function of residue number. (**B**) ^15^N R1 in function of residue number. (**C**) ^15^N R2 in function of residue number. (**D**) R2 over R1 ratios in function of residue number. The solid line in panel (D) corresponds to the average R2/R1 ratio used for obtaining the rotation correlation time τ_c_. The loops Leu12-Gly22 and Ile61-Lys70 are highlighted in all panels.

A per residue view of the conformational dynamics can be obtained from ^15^N R1, R2 and heteronuclear NOEs, which were measured for the 100 non-overlapping cross peaks of *Sa*MazF (Figure 8). Besides the N- and C-termini, low NOE values and especially elevated R1 values (Figure 8A and B) are observed for the residues located in two loops: residues Leu12-Gly22 (S1-S2) and residues Ile61-Lys70 (S4-S5), indicating increased mobility at the ps to sub-ns timescale. Some residues outside these two loop regions show elevated R2 values (Figure 8C), which are indicative of conformational exchange on the microsecond to millisecond timescale (85).

The high RMSD values mentioned earlier for the loop regions Leu12-Ser18 and Lys64-Lys70 in the NMR ensemble and plotted in Figure 5 correlate well with these observations and with an increased flexibility reflected by the decrease in R2/R1 values (and also the N- and C-termini) (Figure 8D). They correlate, however, also with a lower number of long-distance restraints (Supplementary Figure S5). The enhanced conformational flexibility of loop Gly48-Lys54 cannot be deduced from this analysis due to lack of data. It is, however, also prominent in the X-ray ensemble and therefore is likely to be a true feature of *Sa*MazF rather than an artifact of data paucity.

A further global picture of the dynamics of *Sa*MazF can be obtained from the rotational correlation time τ_c_. Analysis of the relaxation data of *Sa*MazF using TENSOR2 (78) indicates an average ^15^N R2/R1 ratio in the most ordered regions of 23.73 (Figure 8D), corresponding to an apparent rotational correlation time τ_c_ of 15.4 ns. The estimated correlation time for a globular protein of the same molecular weight (29.584 kDa) at 308 K is 14 ns (http://nickanthis.com/tools/tau). The slightly higher τ_c_ derived from the R2/R1 ratio is likely due to the two highly flexible termini that increase the effective radius of gyration.

### Dynamics probed by X-ray crystallography

**Figure 9.**
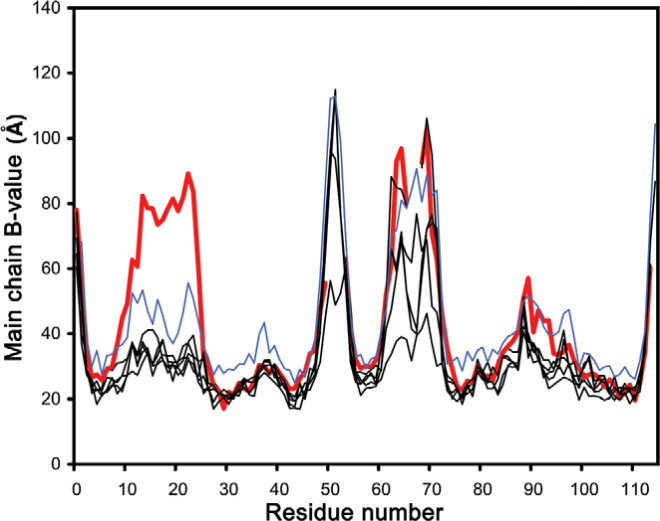
B-factor-derived dynamics. The average backbone B-factors are plotted in function of residue number for all six crystallographic independent monomers from crystal forms I and II and for monomer A of crystal form III. The B-factors in the latter crystal were restrained using non-crystallographic symmetry due to the lower resolution of the data and the profiles for monomers B–H are essentially identical to that of A and therefore not shown. They are in general slightly higher than those for the six monomers from crystal forms I and II over the whole residue range and therefore highlighted in blue. The thick red curve corresponds to monomer B from crystal form I and shows elevated values for residues belonging to loop S1-S2.

Besides structural variation, X-ray crystallography further provides (limited) information on protein dynamics via the atomic B-factors. Variation of the main chain B-factors closely follows the per residue pair-wise RMSD values. There is however one notable exception: in chain B of crystal form I, elevated B-factors are also observed for residues Ala10-Val23 (Figure 9). This is the only indication in our set of crystallographic data that hints toward flexibility of this loop, which in the NMR data behaves as the most dynamic part of the molecule if the termini are excluded.

### *Sa*MazE binding site

**Figure 10.**
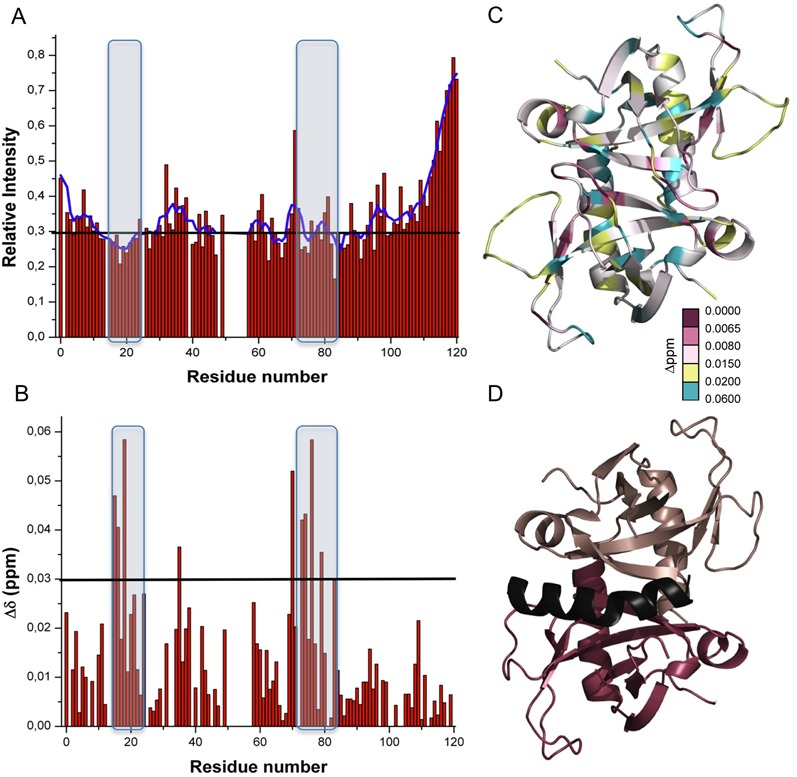
MazE binding. (**A**) Relative change of ^1^H–^15^N HSQC cross-peak intensities in function of residue number upon titration of *Sa*MazF with *Sa*MazE^23–56^ till a 1:1 ratio. The blue curve corresponds to average intensity changes using a sliding window of five residues. Loop S1-S2 and strands S5 and S6 are highlighted. (**B**) Combined ^1^H and ^15^N chemical shift differences between free and bound *Sa*MazF in a 1:1 ratio with *Sa*MazE^23–56^. Loop S1-S2 and strands S5 and S6 are highlighted. (**C**) Combined ^1^H–^15^N chemical shift differences plotted on a ribbon diagram of the *Sa*MazF dimer. Residues are color-coded according to the change in chemical shift of their ^1^H–^15^N HSQC cross-peaks with red corresponding to the largest effects. The orientation is identical to the left panel in Figure 4B. (**D**) Equivalent view of the *B. subtilis* YdcE–YdcD (PDB entry 4ME7) complex. The two YdcE monomers are shown in salmon and red. Residues Met64-Glu83 of the bound antitoxin YdcD are colored black. The N-terminal domain of YdcD is omitted for clarity. Figure created in PyMol (84).

In order to determine the binding site of *Sa*MazE on *Sa*MazF, we performed NMR chemical shift mapping using *Sa*MazE^23–56^, a *Sa*MazE-derived peptide consisting of residues Met23-Glu56. In these experiments, 0.5 mM ^13^C^15^N *Sa*MazF was titrated with 3.5 mM *Sa*MazE^23–56^ up to a final molar ratio of *Sa*MazF_2_:*Sa*MazE^23–56^ of 1:2. The effect of *Sa*MazE^23–56^ mainly consists of a weakening of most of the ^1^H–^15^N HSQC peaks of *Sa*MazF (except for the flexible N- and C-termini) with only small shifts in resonances. As aggregation was observed at the end of the titration, we based our analysis on the fifth titration point corresponding to a 1:1 ratio. Figure 10A and B plots the effects of *Sa*MazE^23–56^ on the intensities and chemical shifts of the ^1^H–^15^N HSQC cross-peaks. Although the statistical reliability is limited, the largest effects for chemical shift changes are found in loop S1-S2 and strands S5 and S6, which makes sense in terms of the toxin-antitoxin interactions observed in the related YdcE–YdcD complex (86) (Figure 10C and D and Supplementary Figure S7). Loop S1-S2 needs to move to an open conformation to allow antitoxin binding in YdcE. Strand S6 is located underneath loop S1-S2 and is a major part of the interaction surface for YdcD residues Met64-Glu83, the segment that corresponds to our *Sa*MazE^23–56^ peptide. Within the MazF subfamily to which *Sa*MazF belongs, the residues involved in antitoxin and substrate binding are well conserved (Supplementary Figure S1). In addition, CD measurements indicate that *Sa*MazE^23–56^ adopts an α-helical conformation when bound to *Sa*MazF (data not shown). These observations are in agreement with a conserved mode of inhibition within the *mazEF* modules.

## DISCUSSION

Because of their biochemical activities that often lead to cell death upon over-expression, wild-type TA toxins can usually only be expressed in presence of their cognate antitoxin and therefore are difficult to obtain in large quantities. Indeed, production of *E. coli Ec*MazF of suitable quality and quantity for structural studies was reported to require a mutation that abrogates its RNase activity (87). Attempts to purify wild-type *Ec*MazF in presence of the antitoxin *Ec*MazE using an unfolding/refolding protocol (17) led to protein with a low solubility and a poor NMR spectrum (87).

To overcome these problems, we designed an on-column separation protocol that allows separating *Sa*MazF from *Sa*MazE without compromising protein quality. Likely our approach was facilitated by the biophysical properties of *Sa*MazF. Unfolding of *Sa*MazF is kinetically limited and aggregation-driven. Possibly only a small fraction of *Sa*MazF (if any) unfolds during the procedure used to strip *Sa*MazE from the Ni-NTA-bound *Sa*MazF. As aggregation is not possible because the *Sa*MazF dimers remain physically separated from each other on the column during the removal of *Sa*MazE, a high yield of well-folded *Sa*MazF is possible.

Whether or not *Sa*MazF (partially) unfolds during the on-column separation protocol is difficult to establish. Guanidinium-induced unfolding of *Sa*MazF cannot be followed by fluorescence spectroscopy as the protein does not posses tryptophan and its four tyrosine side chains are fully solvent exposed. CD measurements in 3 M GdHCl are not possible below 220 nm. While the CD spectrum of *Sa*MazF incubated for 5 h in 3 M GdHCl is, within the margins of error, identical to that of *Sa*MazF in absence of GdHCl, this cannot be taken as a proof of lack of unfolding or dissociation into monomers. DLS measurements are hampered by the difference in viscosity of the solutions, making it difficult to compare hydrodynamic radii. Control experiments using colloidal gold nano-particles (Nanopartz) indicate a correction factor of 1.5 to the hydrodynamic radius for the use of 3 M GdHCl, and when applying this correction factor, the hydrodynamic radius of *Sa*MazF remains unaltered upon 1 h of exposure to 3 M GdHCl.

Thermal unfolding of *Sa*MazF contrasts with the two-state unfolding of F-plasmid and *Vibrio fischeri* CcdB, two proteins that share the same tertiary and quaternary structure (88,89). Unfolding of *Sa*MazF monomers is kinetically limited and even at temperatures higher than 363 K the monomers do still have an appreciable lifetime. Unfolding leads to rapid aggregation into large particles with a large amount of β-structure. Possibly the high activation energy for unfolding of the *Sa*MazF monomer was selected to prevent aggregation of *Sa*MazF *in vivo*. Indeed, at physiological temperatures (*T* < 313 K), unfolding and therefore aggregation is highly unlikely to occur.

Although overall highly similar, the X-ray- and NMR-derived structures represent distinct conformational ensembles and distinct profiles of backbone dynamics. In the X-ray ensemble, conformational variability and dynamics is mainly located in loop Ile61-Lys70 (between strands S4 and S5) and to a lesser extent in loop Gly48-Lys54 (between strands S3 and S4). The NMR ensemble on the other hand shows increased dynamics and structural variability in loops Leu12-Ser18 (between strands S1 and S2), and Gly48-Lys54, and less pronounced in loop Lys64-Lys70. Of these, the backbone dynamics of loop Lys64-Lys70 is likely not of direct functional importance. The other two loops on the other hand change conformation between the substrate- and antitoxin-bound states in the closely related YdcE (86). In this respect, the NMR ensemble and its ^15^N relaxation-derived backbone dynamics correlate better with the proposed molecular mechanisms behind MazF regulation (86). The importance of dynamics in loop Leu12-Ser18 can in the X-ray ensemble only be inferred from one out of 14 monomers (form I chain B), where this loop shows elevated B-factors. Not surprisingly, in this monomer, the loop is not involved in lattice contacts. In general, it appears that loops S1-S2, S3-S4 and S4-S5 have a preferred conformation which can be modulated by ligand binding. The latter potential for conformational change is further reflected in crystal-packing mediated loop conformations and in the NMR order parameters. The individual conformations of these loops as well as the larger structural variation present in the NMR ensemble are probably for the larger part due to lack of sufficient NOE restraints while differences between the X-ray and NMR ensembles due to crystal packing interactions are restricted to loop S4-S5 and to a smaller extent to loop S3-S4.

While the NMR data seem to be able to indicate more correctly which loops may undergo functional dynamics during ligand binding (both RNA and MazE), neither crystallography nor NMR provide information on the actual conformations that are to be adopted in the bound states. For each of the three dynamic loops, the NMR ensemble shows a single conformational family that each time encompasses the most populated conformational family observed in the X-ray ensemble. The alternative conformations observed in the X-ray ensemble for loops S4-S5 on the other hand are not related to conformations observed in the RNA- or MazE-bound forms of the closely related YdcE (86).

When comparing with other MazF family members with known structure, *Sa*MazF has its highest sequence identity with YdcE from *Bacillus subtilis* (64%) (Supplementary Figure S1), which is reflected in an RMSD of 0.73 Å for 110 common Cα atoms and which deviates in structure mainly in the conformation between Gly48 and Ile55, a region that is also conformationally heterogeneous within our population of *Sa*MazF monomers. Sequence identity is much weaker for R1 Kid (22% corresponding to 1.57 Å for 110 Cα atoms) where conformational differences are extended to Glu62-Ser72 and Asp83-Lys90, and for *E. coli Ec*MazF (18% corresponding to 1.69 Å for 95 Cα atoms) where in addition to the already mentioned regions, the loops Leu9-Pro25 and Ile29-Thr40 also adopt different structures. Secondary structure elements are nevertheless well conserved.

The MazF family as a whole is a highly divergent family at the sequence level (Supplementary Figure S1). With the exception of two essential catalytic residues (Arg24 and Thr47), residues implicated in substrate and antitoxin recognition are not specifically conserved, in agreement with the existence of at least two structurally different families of MazF-associated antitoxins (exemplified by the crystal structures of the *E. coli* and *B. subtilis* MazF–MazE complexes). To compare RNA binding and specificity between *Sa*MazF and YdcE, we constructed a model of *Sa*MazF bound to 5′UUdUACAUAA3′ and mapped the amino acid differences between *Sa*MazF and YdcE (Supplementary Figure S6A). Within the vicinity of the two likely catalytic residues Arg24 and Thr47, only one substitution is observed between *Sa*MazF and YdcE: Gln50 of YdcE is replaced by Arg49 in *Sa*MazF (Supplementary Figure S6B). This substitution is neutral with respect to RNA specificity as interactions can only be made with the phosphate backbone. Other substitutions between both proteins involving side chains contacting the bound 9-mer substrate mimic cluster at the 3′ (Thr33, Lys36 and Tyr37) and 5′ (Leu9, Leu68, Asp69, Lys70, Lys88, Glu89 and Leu91) ends and do not affect the core UACAU sequence that seems to be the target of most if not all MazF proteins. The amino acid side chains that are involved in base recognition of the UACAU core sequence (Ser18, Gln20, Thr47, Lys52, Leu55, His58, Phe68, Ser72, Glu77 and Gln78) tend to be well conserved among the closer homologues of *Sa*MazF (35% sequence identity or higher), and for most of them it was shown that alanine substitutions inactivate YdcE (86). The only highly conserved residue that is not involved in RNA recognition (or catalysis) is Asn35. Its side chain is buried in a hydrophilic cluster and seems to have a structural role.

Within the *Sa*MazF subfamily (sequences that show at least 35% sequence identity to *Sa*MazF) residue conservation also correlates well with the NMR mapping of *Sa*MazE^23–56^. In the segment that binds to the toxin, *Sa*MazE and YdcD share 42% sequence identity, while for the residues of YdcE interacting with YdcD, 85% are conserved with *Sa*MazF. Furthermore, superposition of the YdcE–YdcD complex on *Sa*MazF indicates that those residues conserved between *Sa*MazE and YdcD are capable of making identical TA interactions. Thus, although *Sa*MazE is considerably shorter than YdcD, its toxin-neutralizing segment is expected to adopt the same conformation when bound to *Sa*MazF as does YdcD when bound to YdcE.

Protein function not only depends on protein structure but also on dynamics. While the conservation of protein structure during evolution is well established (90), fewer studies are available that examine protein dynamics and its relationship with protein function in an evolutionary context. While there is accumulating evidence that protein dynamics is often evolutionarily conserved (91), conserved activities of related proteins may use distinct dynamic mechanisms (92). We therefore compared the profiles of dynamics of *Sa*MazF to that of *Ec*MazF and to the F-plasmid and *V. fischeri* CcdB proteins, which adopt the same tertiary and quaternary fold (89,93) but function as gyrase inhibitors (21,94). Regions with elevated dynamics in *Ec*MazF as observed by NMR correspond to the same three loops as seen for *Sa*MazF: S1-S2, S3-S4 and S4-S5, with again loop S1-S2 being the most pronounced (87). More interesting however is that the S1-S2 and S3-S4 loops also show pronounced dynamics in *V. fischeri* CcdB (89) and that the S1-S2 loop undergoes a disorder-to-order transition in going from the target-bound structure to the antitoxin-bound structure in F-plasmid CcdB (88). Thus, the pattern of dynamics seems to be conserved within the MazF/CcdB superfamily and exploited in an equivalent way for functionality. While this may be a consequence of a common mode of antitoxin binding, it should be noted that the substrates of the MazF and CcdB proteins are completely unrelated (RNA and gyrase), and that in both cases substrate and antitoxin binding sites only partially overlap. In addition, the disorder-to-order transition in loop S1-S2 occurs in opposite directions in MazF and CcdB (21,23,86,93), suggesting an equivalent exploitation of the dynamic potential but with this mechanism independently acquired in the MazF and CcdB families.

## PROTEIN DATA BANK ACCESSION NUMBERS

Coordinates have been submitted to the Protein Data Bank with accession numbers 2MF2, 4MZM, 4MZP and 4MZT.

## SUPPLEMENTARY DATA

Supplementary Data is available at NAR Online.

SUPPLEMENTARY DATA
