# Supplementary material for: Structural and biophysical characterization of Staphylococcus aureus SaMazF shows conservation of functional dynamics
Source: Nucleic Acids Res. 2014 Apr 19;42(10):6709–25. doi: 10.1093/nar/gku266 (PMC4041440; doi:10.1093/nar/gku266)
Supplement: SUPPLEMENTARY DATA [file supp_42_10_6709__index.html]

Structural and biophysical characterization of Staphylococcus aureus SaMazF shows conservation of functional dynamics — Structural and biophysical characterization of Staphylococcus aureus SaMazF shows conservation of functional dynamics — SUPPLEMENTARY DATA 

# Structural and biophysical characterization of *Staphylococcus aureus Sa*MazF shows conservation of functional dynamics

## SUPPLEMENTARY DATA

**Files in this Data Supplement:**

- SUPPLEMENTARY DATA
