# Supplementary material for: Structural and biophysical characterization of Staphylococcus aureus SaMazF shows conservation of functional dynamics
Source: Nucleic Acids Res. 2014 Apr 19;42(10):6709–25. doi: 10.1093/nar/gku266 (PMC4041440; doi:10.1093/nar/gku266)
Supplement: SUPPLEMENTARY DATA [file supp_gku266_Supplementary_Fig_FINAL_02042014.pdf]

Supplementary Material

Supplementary Figure 1

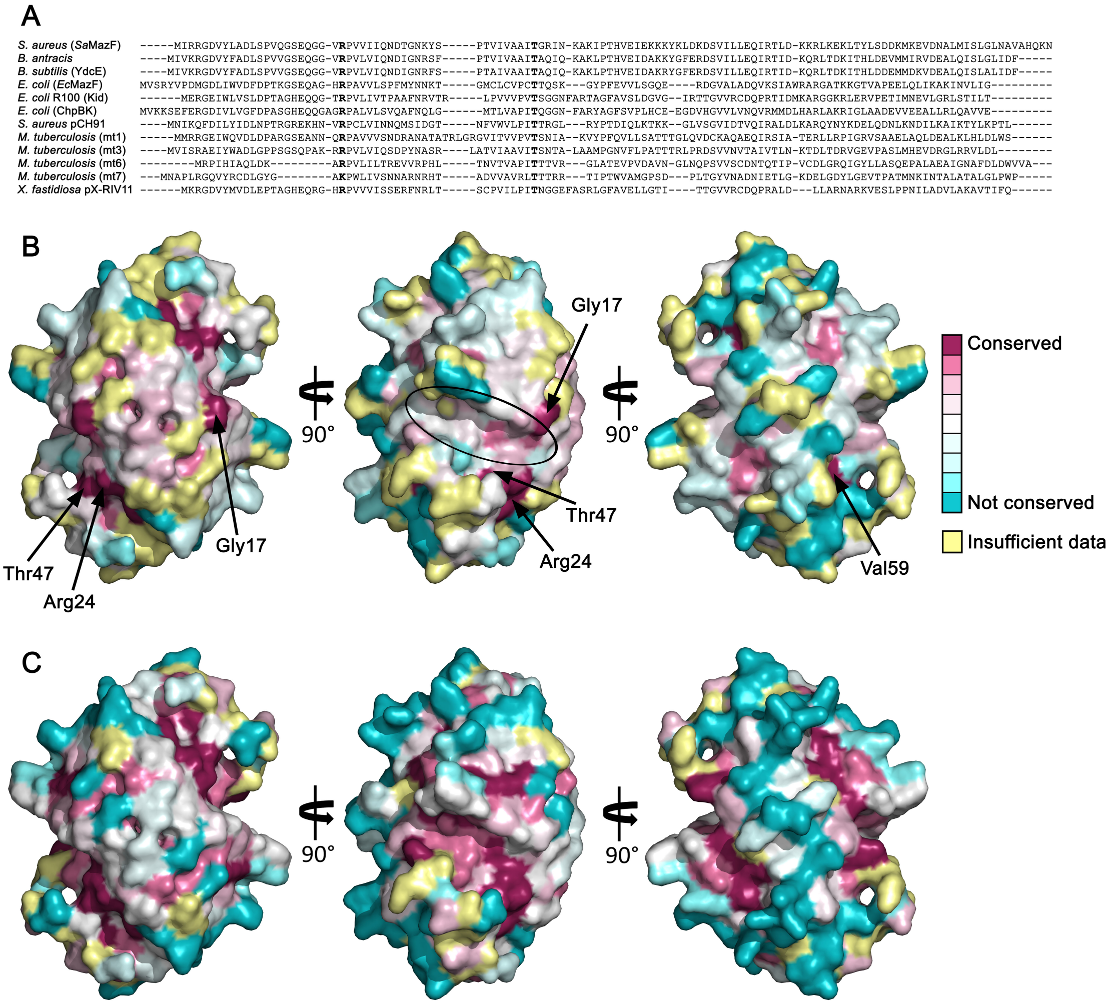

Supplementary Figure S1. Residue conservation within the MazF family. A. Amino acid sequence alignment of 12 MazF family members for which the mRNA interferase activity was confirmed and the cutting sequences determined. The alignment is adapted from (Simanshu et al., 2013) with minor manual corrections to avoid insertions or deletions in the main secondary structure elements that are conserved in the crystal structures of *Sa*MazF, YdcE, *Ec*MazF and Kid. The sequences of the MazF homologs from *S. aureus* plasmid pCH91 and from *X. fastidiosa* plasmid pX-RIV11 were added manually. The catalytically important residues Arg24 and Thr47 are shown in bold. **B.** Sequence conservation within the MazF family mapped on the surface of *Sa*MazF. Conservation scores were calculated using ConSurf (Ashkenazy et al., 2010) using the alignment shown in panel A and plotted on the surface of the *Sa*MazF dimer. Orientations are identical as in Figure 4B. The most highly conserved residues are indicated and consist of the two catalytic residues Arg24 and Thr47 as well as a few residues that probably have a structural role. The remainder of the residues

implicated in substrate and antitoxin recognition are not specifically conserved, in agreement with the existence of at least two structurally different families of MazF-associated antitoxins (exemplified by the crystal structures of the *E. coli* and *B. subtilis* MazF-MazE complexes (Kamada et al., 2003; Simanshu et al., 2013). The RNA/MazE binding groove is highlighted using an ellipse. **C.** Equivalent views of the *Sa*MazF dimer, but color-coded according to residue conservation. Conservation scores were calculated using ConSurf based on 12 sequences that show between 18 and 64% sequence identity with *Sa*MazF (red most conserved and blue least conserved). Residues where the calculated conservation scores are statistically meaningless are colored yellow. Both residues involved in substrate binding and antitoxin binding show the highest conservation scores. Figure prepared using PyMol (PyMOL Molecular Graphics System, Version 0.99 Schrödinger, LLC).

Supplementary Figure 2

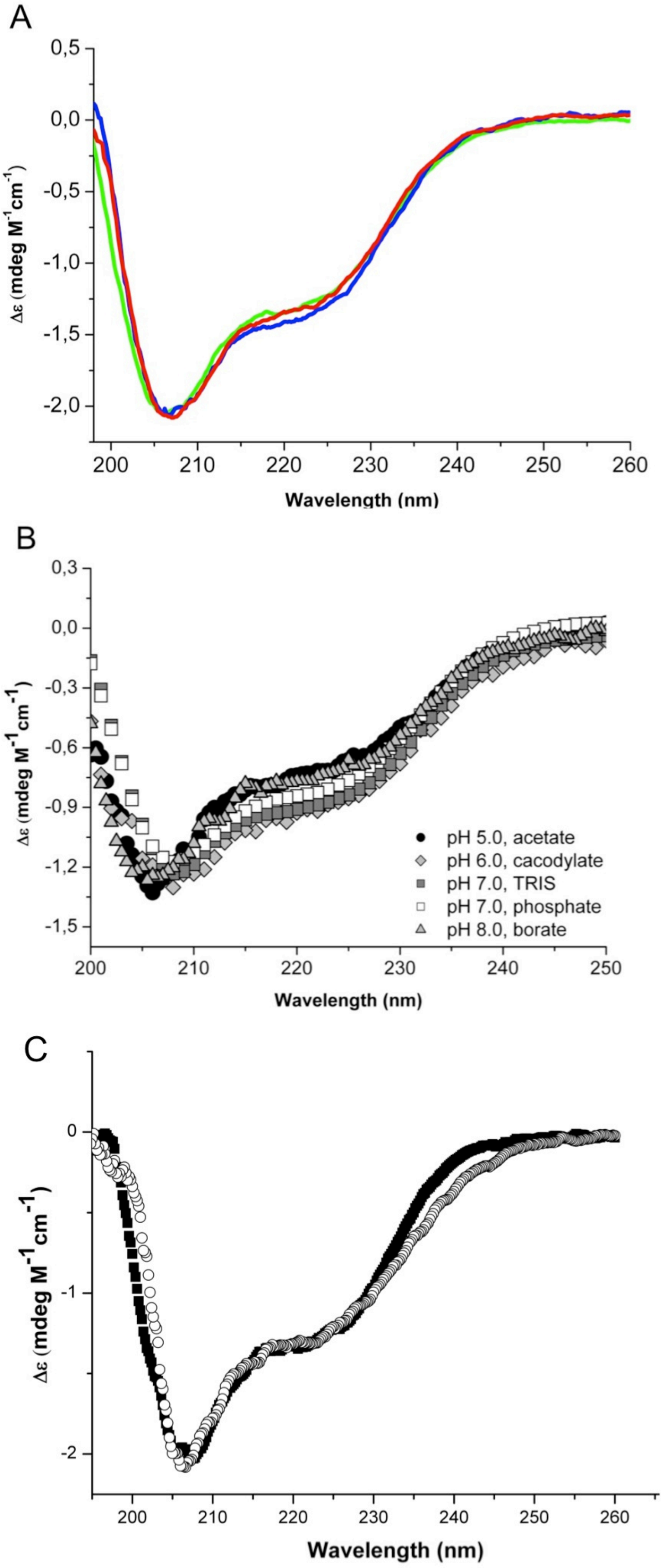

Supplementary Figure S2. Biophysical characterization. A. Circular dichroism spectra of *Sa*MazF at 293 K in 20 mM TRIS-HCl buffer pH 7.0 and different concentrations of NaCl (0 mM NaCl green, 75 mM NaCl blue, 300 mM NaCl red). The three spectra are essentially identical to the corresponding ones collected in phosphate buffer and shown in Figure 2C. **B.** Circular dichroism spectra of *Sa*MazF at 293 K in different buffer conditions: 20 mM Na-phosphate pH 7.0, 75 mM NaCl, 20 mM TRIS-HCl pH 7.0, 75 mM NaCl, 20 mM Na-acetate pH 5.0, 75 mM NaCl, 20 mM Na-cacodylate pH 6.0, 75 mM NaCl, 20 mM Na-borate pH 8.0, 75 mM NaCl. All the spectra are reasonably equivalent to each others showing that *Sa*MazF remains well-folded with its typical secondary structure through a wide range of buffers and pHs. **C.** Circular dichroism spectrum of *Sa*MazF in 20 mM TRIS-HCl pH 7.0, 75 mM NaCl at 293 K (black squares) and immediately after heating at 371 K (open circles). Differences between both spectra are minor and indicate absence of any major structural rearrangement or unfolding process within the time of the experiment.

Supplementary Figure 3

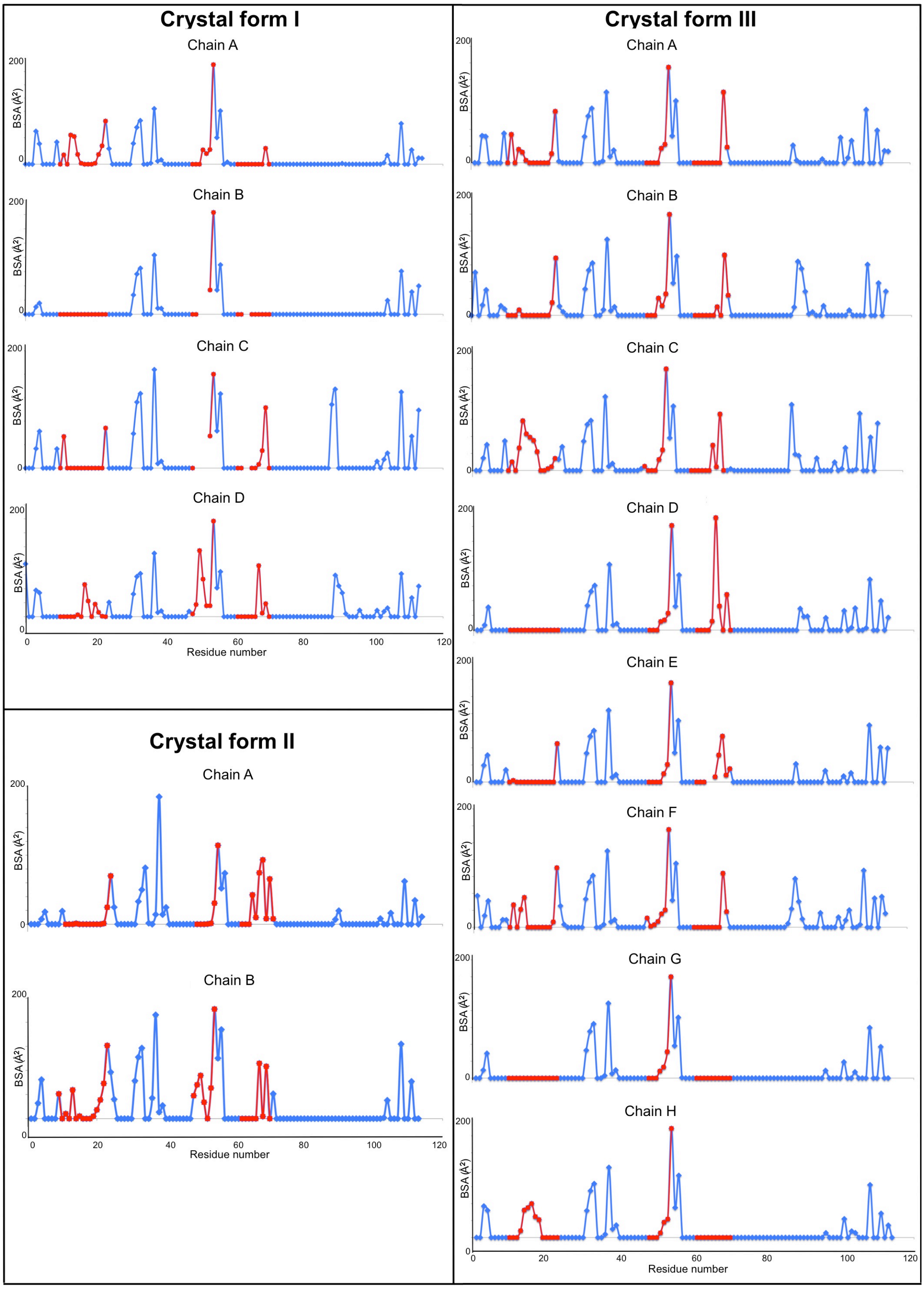

Supplementary Figure S3. Crystal packing. The plots summarize in function of amino acid number the amount of surface area buried in crystal lattice contacts for every chain. Loops S1-S2, S3-S4 and S4-S5 are highlighted in red. It is clear that lattice contacts are not

randomly distributed on the protein surface. Indeed loops S3-S4 and S4-S5 are most often involved in crystal packing.

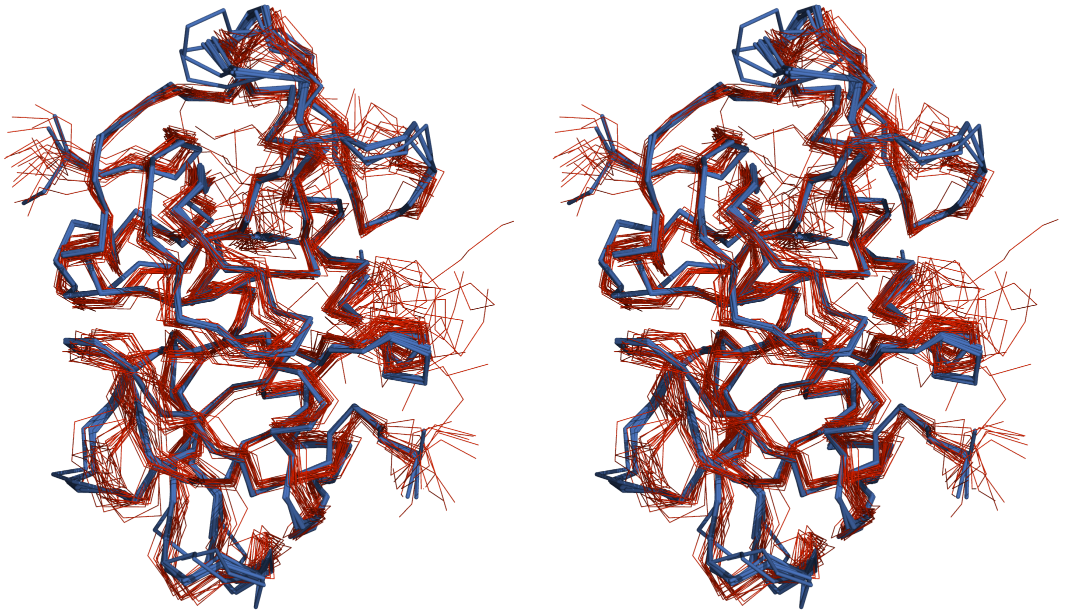

Supplementary Figure S4. Comparison between the X-ray and NMR ensemble. The seven independently determined crystallographic dimers are shown in blue while the 20 lowest energy NMR dimers are shown in red. The highly flexible N-terminal (His-tag) and C-terminal tails are removed from the NMR ensemble for clarity. It can be seen that the X-ray ensemble is tighter and that the major differences between both ensembles are located in loops S1-S2, S3-S4 and S4-S5, corresponding to Leu12-Ser18, Gly48-Thr57 and Ile61-Ser72 respectively. Figure prepared using PyMol (PyMOL Molecular Graphics System, Version 0.99 Schrödinger, LLC).

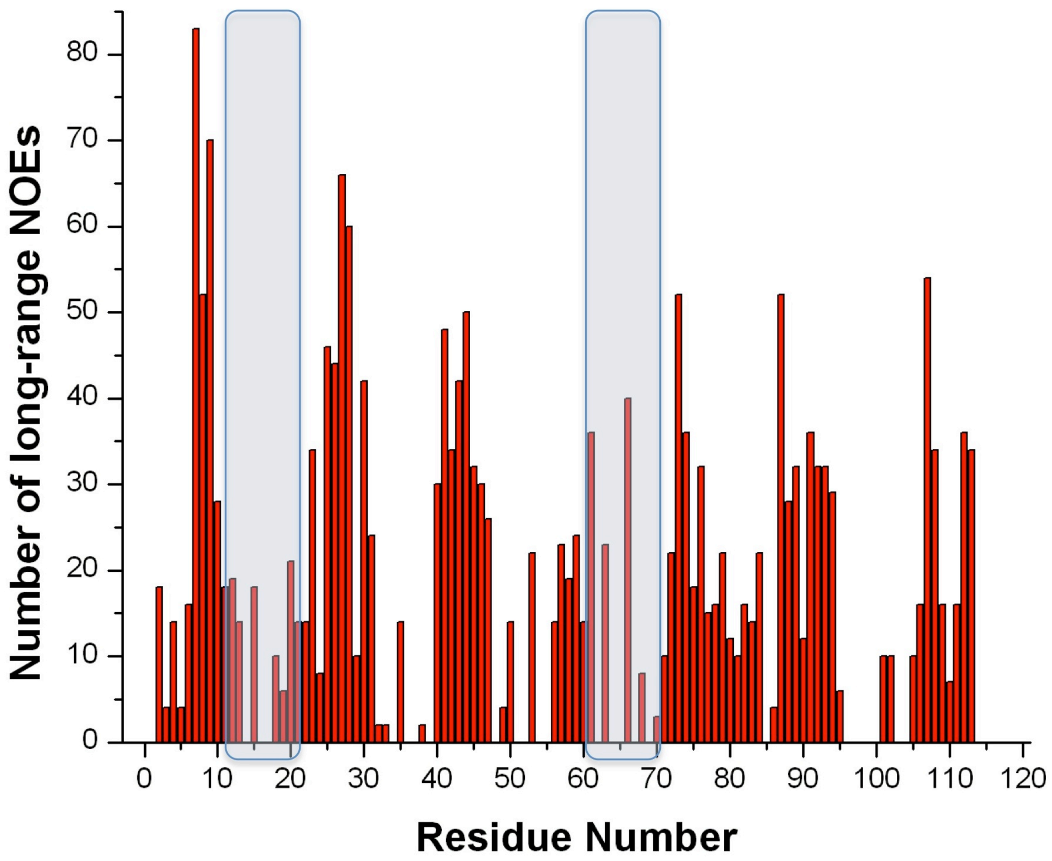

Supplementary Figure S5. Number of long-range nuclear Overhauser enhancements (NOEs) versus residue number. NOEs are time- and ensemble-averaged parameters, which were used for better exploring both structural and dynamical features. Increased mobility in loops Leu12-Gly22 and Ile61-Asp70 is confirmed by low number of NOEs (highlighted bands), manifesting elevate conformational flexibility.

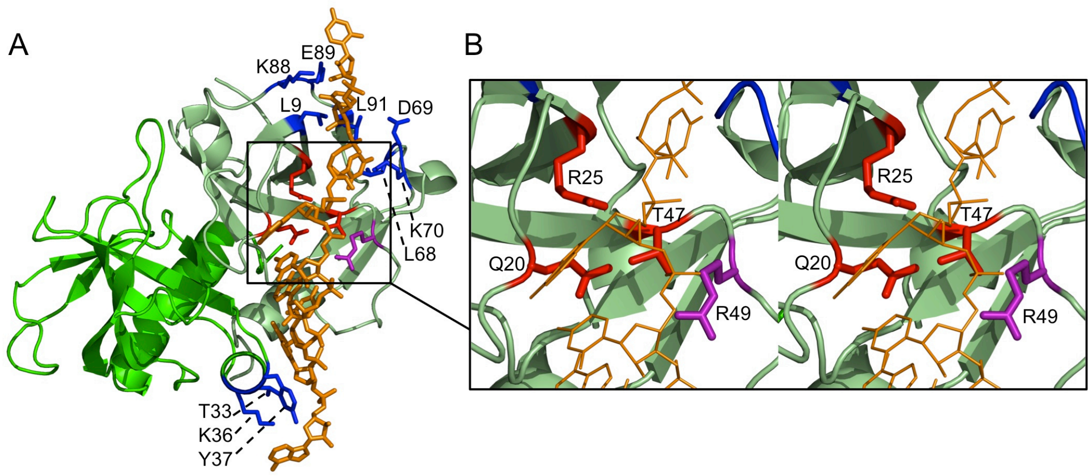

Supplementary Figure S6. MazF-RNA model. A. Model of the *Sa*MazF bound to 5'UUdUACAUAA3'. The two *Sa*MazF monomers are depicted in different shades of green. Amino acids substituted between *Sa*MazF and YdcE are shown as blue sticks, except for Arg49 which is shown in purple. The two catalytic residues (Arg25 and Thr47) and the conserved Gln20 are shown in red. **B.** Zoom-in view (stereo) of the conserved catalytic site. The important residues are labeled and drawn as red sticks. The Gln50-Arg49 substitution from YdcE to MazF is neutral with respect to RNA specificity and shown in purple. Figure prepared using PyMol (PyMOL Molecular Graphics System, Version 0.99 Schrödinger, LLC).

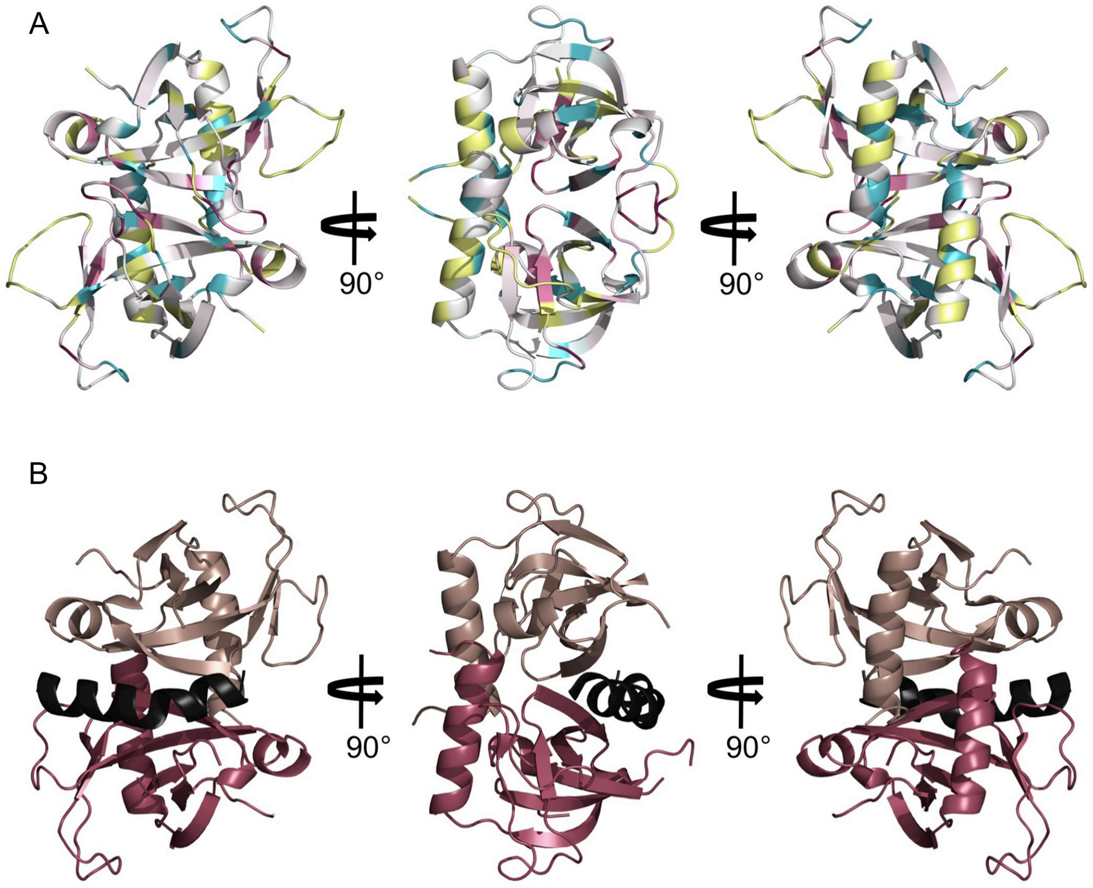

C

*Sa*MazE ---MLSF**S**QNRSHSLEQSLKE**GY**SQ**MA**DL**NL**SLAN**EA**FPI**ECEA**CDCN**E**TYL**S**SNSTNE
*Bs*YdcD MSESSARTEMKISLPENLVAELDGVAMREKRSRNELISQAVRAYVFT**S**ERTTRHNRDLMRR**GY**ME**MA**KI**NL**NISS**EA**HFA**ECEA**ETTV**E**RLV**S**GG----

Supplementary Figure S7. Interaction with *Sa*MazE. A. Combined ^{1}H-^{15}N chemical shift differences plotted on a ribbon diagram of the *Sa*MazF dimer. Residues are color-coded according to the change in chemical shift of their ^{1}H-^{15}N HSQC cross-peaks with red corresponding to the largest effects (see Figure 10 for the exact scale). Orientations are identical to those in Figure 4B. **B.** Equivalent views of the *B. subtilis* YdcE-YdcD (PDB entry 4ME7) complex. The two YdcE monomers are shown in salmon and red. Residues Met64-Glu83 of the bound antitoxin YdcD are colored black. The N-terminal domain of YdcD is omitted for clarity. **C.** Amino acid sequence alignment of *Sa*MazE and *Bs*YdcD. Panels A and B were created in PyMol (PyMOL Molecular Graphics System, Version 0.99 Schrödinger, LLC).

Supplementary References

Simanshu,D.K., Yamaguchi,Y., Park,J.H., Inouye,M., and Patel,D.J. (2013) Structural basis of mRNA recognition and cleavage by toxin MazF and its regulation by antitoxin MazE in *Bacillus subtilis*. *Mol*. *Cell*, **52**, 447-458.

Ashkenazy,H., Erez,E., Martz,E., Pupko,T., and Ben-Tal,N. (2010) ConSurf 2010: calculating evolutionary conservation in sequence and structure of proteins and nucleic acids. *Nucleic Acids Res.,* **38**, W529-W533.

Kamada K., Hanaoka,F., and Burley,S.K. (2003) Crystal structure of the MazE/MazF complex: molecular bases of antidote-toxin recognition. *Mol. Cell,* **11**, 875–884.

The PyMOL Molecular Graphics System, Version 0.99 Schrödinger, LLC.
